# Selective Elimination of Genetic Variants of Human Embryonic Stem Cells from High Vulnerability to Ferroptosis

**DOI:** 10.34133/bmr.0093

**Published:** 2024-10-25

**Authors:** Yun-Jeong Kim, Seon Min Kim, Young-Hyun Go, Han Sun Kim, Sunghyouk Park, Yun Pyo Kang, Hyuk-Jin Cha

**Affiliations:** ^1^ College of Pharmacy, Seoul National University, Seoul, Republic of Korea.; ^2^ Natural Products Research Institute, College of Pharmacy, Seoul National University, Seoul, Republic of Korea.; ^3^ Research Institute of Pharmaceutical Sciences, Seoul National University, Seoul, Republic of Korea.

## Abstract

Despite the great promise of human pluripotent stem cell (hPSC)-based cell therapy, safety concerns arise from genetic aberrations during in vitro culture, due to their uncertain consequences. Notably, these genetic aberrations confer a survival trait known as “culture-adaptation”, allowing aberrant hPSCs to evade apoptosis and outcompete normal cells. Thus, it is crucial to develop strategies for selectively eliminating aberrant hPSCs to ensure the safety of therapeutic applications. Herein, we discovered that hPSCs with genetic variations exhibited increased glycolysis and active fatty acid biosynthesis. Surprisingly, these variants, showing resistance to stress-induced apoptosis, were paradoxically susceptible to ferroptosis by the treatment of RAS-selective lethal 3 (RSL3), a glutathione peroxide 4 inhibitor. The selective sensitivity to RSL3 resulted from elevated levels of polyunsaturated fatty acids containing phospholipids, driven by the up-regulation of acyl-coenzyme A synthetase long-chain family member 4 through Yes1-associated protein 1 activity. Importantly, the distinct sensitivity of normal hPSCs and metabolic variants to ferroptosis enabled the targeted removal of genetically aberrant hPSCs through RSL3 treatment, while normal hPSCs transiently exposed to RSL3 maintained their pluripotency and normal differentiation capacity. These findings hold important promise for the maintenance of genetically normal hPSCs during extended in vitro culture, thereby ensuring the safety and efficacy of hPSC-based cell therapies.

## Introduction

In recent years, substantial progress has been made in the clinical application of human pluripotent stem cell (hPSC)-based cell therapy for degenerative diseases. However, safety concerns associated with hPSC-based therapies, particularly tumorigenesis, remain critical technical obstacles [1]. Various strategies have been developed to prevent teratoma formation by residual undifferentiated hPSCs [2,3]. Nevertheless, genetic aberrations that occur during in vitro culture of hPSCs, such as somatic mutations like tumor protein P53 (TP53) mutations [4], copy number variations such as gain at 20q11.21 [5], and chromosomal alterations [6], are considered serious risk factors due to the clinical uncertainties they introduce [7]. One distinct consequence of genetically aberrant hPSCs is the acquisition of a survival trait often referred to as “culture adaptation” [5]. This adaptation allows these cells to evade the highly efficient apoptotic mechanisms [8] that typically eliminate naturally occurring mutants and maintain genetic integrity [9]. Dominant-negative mutations in TP53 [4], induction of *BCL2L1* [8], or high Yes1-associated protein 1 (YAP1) activity [10,11], cellular events frequently observed in “culture-adapted” hPSCs, contribute to the acquisition of this survival trait, enabling genetic variants to outcompete normal hPSCs and become dominant over time [4,11]. Furthermore, the escape of aberrant mitotic cells from cell death has been suggested as a cause of further genetic aberrations [12]. Therefore, early detection or selective elimination of genetically aberrant hPSCs is critical to ensure the safety of hPSC-based clinical applications [13].

hPSCs with primed pluripotency primarily rely on glycolysis for adenosine triphosphate (ATP) production [14,15], resulting in a high demand for glucose and glutamine and elevated lactate production [16]. Additionally, de novo fatty acid synthesis is crucial for maintaining pluripotency [17] and the survival of hPSCs [18]. Knockout (KO) of genes involved in fatty acid synthesis, such as fatty acid synthase (*Fasn*) [19] or acetyl-coenzyme A (CoA) carboxylase 1 (*Acc1*) [20], results in embryonic lethality, highlighting the critical roles of fatty acids in normal embryo development. The requirement for beta-oxidation of fatty acids (or fatty acid oxidation [FAO]) in early embryo development is recapitulated in the survival of embryonic stem cells (ESCs) [21] and the reprogramming of induced pluripotent stem cells (iPSCs) [22]. Notably, unsaturated fatty acids (UFAs) and their metabolites are abundant in ESCs [23]. Specifically, during cellular reprogramming, the level of monounsaturated fatty acids (MUFAs) increases, while the level of polyunsaturated fatty acids (PUFAs) decreases [24]. Stearoyl-CoA desaturase, the rate-limiting enzyme responsible for converting saturated fatty acids to MUFAs, is uniquely essential for human ESC (hESC) survival [25]. The essentiality of stearoyl-CoA desaturase activity in ESCs has been demonstrated by the addition of oleic acid (a MUFA) in ESC culture medium, whereas the inhibition of PUFA biosynthesis maintains stemness [26].

Our findings revealed that hESCs with genetic variations, specifically, the gain of 20q11.21 and TP53 mutation, and a survival trait characterized by high YAP1 activity and *BCL2L1* induction, exhibit metabolic alterations. These alterations include increased glycolysis and active fatty acid biosynthesis. Surprisingly, these metabolic variants, which are resistant to stress-induced apoptosis, are highly susceptible to ferroptosis induced by RAS-selective lethal 3 (RSL3), a potent glutathione peroxide 4 (GPX4) inhibitor. Here, we identified the YAP1-dependent induction of acyl-coenzyme A synthetase long-chain family member 4 (ACSL4) with high levels of phospholipids containing PUFAs as the underlying cause of ferroptosis vulnerability in the metabolic variants. Importantly, the distinct sensitivity of the metabolic variants to ferroptosis allows for the selective elimination of these variants (i.e., genetically aberrant hESCs) through RSL3 treatment. When exposed to RSL3 for only a brief period, normal hESCs maintain their pluripotency and ability to differentiate normally. These findings suggest that this selection approach could be valuable for maintaining genetically normal hESCs during long-term in vitro culture.

## Materials and Methods

### Cell culture

hESC (WA09: H9, WiCell Research Institute, CHA3-hESCs) and human iPSC lines (BJ-iPSCs) were maintained in iPSC-brew MACS (Miltenyi Biotec, #130-104-368) with 0.1% gentamycin (Gibco, Waltham, MA, USA, #15750-060) on a Matrigel (Corning, Corning, NY, USA, #354277)-coated cell culture dish at 37 °C and humidified to 5% in a CO_2_ incubator. Upon transfer, hPSCs were rinsed with Dulbecco’s phosphate-buffered saline and detached enzymatically with Dispase (Life Technologies). Y27632 (10 μM; PeproTech #1293823) was used for 1 d after the attachment if needed.

### Reagents

The primary antibodies against α-tubulin (#sc-8035), β-actin (#sc-47778), YAP1 (#sc-101199), Vinculin (sc-25336), and ACSL4 (sc-271800) were purchased from Santa Cruz Biotechnology, Inc. Antibodies against B-cell lymphoma-extra large (BCL-xL) (ab32370) were from Abcam. The secondary antibodies against mouse immunoglobulin G (#115-035-003) and rabbit immunoglobulin G (#111-035-003) were purchased from Jackson ImmunoResearch Laboratories, Inc.

### Gene set enrichment analysis and Kyoto Encyclopedia of Genes and Genomes analysis

Gene set enrichment analysis (GSEA) was performed using the microarray dataset (GSE9709, GSE16963, and GSE119386), obtained from the Gene Expression Omnibus database (https://www.ncbi.nlm.nih.gov/geo/) as described previously [27]. The GSE9709 and GSE16963 are microarray datasets from the isogenic pair of hESC and the differentiated cells and were divided into 2 groups as indicated. The ranks for differently expressed RNA/genes between hESCs and differentiated counterparts were yielded by GEO2R (https://www.ncbi.nlm.nih.gov/geo/geo2r/). The inhouse geneset, GSE119386, includes the read-count result of RNA sequencing of normal hESCs and variant hESCs, was divided into 2 groups. Differentially expressed gene (DEG) of the dataset was analyzed and acquired by “DEseq” and “vst” functions of R package. Using each DEG list as input, GSEA analysis was performed using the gene sets related to fatty acid synthesis from MSigDB via the R package “fgsea”. By using in-house single-cell RNA sequencing data, DEGs were selected based on logFC>1 and adjusted *P* value < 0.05. Using DEGs as an input, we performed Enrichr (https://maayanlab.cloud/Enrichr/) analysis. The Kyoto Encyclopedia of Genes and Genomes pathway analysis was performed for the validation of prediction using inhouse bulk RNA sequencing data by the R package “pathview” (https://doi.org/10.1093/bioinformatics/btt285). Significantly up-regulated or down-regulated Gene Ontology terms were selected with adjusted *P* value < 0.05 and a |normalized enrichment score| > 1.4.

### Cell death, clonogenic assay, and live cell imaging

Cell death was collected by flow cytometry (FACS Calibur and Celesta, BD Bioscience), after 1-h Annexin V (BD Bioscience, Franklin Lakes, NJ, USA, #556419) and 7-aminoactinomycin D (7-AAD) (BD Bioscience, #559925) staining. For a clonogenic assay, 1 × 10^3^ cells were briefly cultured in 6-well plates and treated with indicted doses of chemicals every 1 d. Exact dose and time of the experiments are listed in the legend of each figure. Live cell imaging for monitoring cell death was conducted by JuLI Stage (NanoEnTek Inc.) and analyzed with JuLI STAT software as per the manufacturer’s instruction. The cell image of the bright field was captured by a light channel optical microscope (Olympus, Tokyo, Japan, CKX-41) or JuLI Stage (NanoEntek, Seoul, Korea).

### Nuclear magnetic resonance for fatty acid quantification

The harvested cell pellets underwent standard 2-phase extraction, and both phases were separated and dried using SpeedVac. The dried samples from lipid phase were dissolved in chloroform-d_6_ (Sigma-Aldrich), and 1-dimensional (1D) proton nuclear magnetic resonance (NMR) was taken using an 800-MHz Bruker Avance III HD spectrometer equipped with a 5-mm CPTCI CryoProbe (Bruker BioSpin, Germany). The dried samples from aqueous phase were dissolved in D_2_O with 5 mM NaH_2_PO_4_, 2 mM Na_2_HPO_4_, and 0.025% trimethylsilylpropanoic acid, and 1D proton nuclear overhauser enhancement spectroscopy NMR was taken using the same spectrometer. The number of scans was 32. For the quantification of the fatty acids, the area under the spectrum of the regions of lipid phase sample (PUFAs [2.7 to 2.9 ppm], total UFAs [5.305 to 5.42 ppm], and total fatty acids [1.2 to 1.4 ppm]) was normalized by 0.6 to 4.0 ppm of the respective aqueous phase sample.

### Lipidomics analysis

Cells (4 × 10^6^ per sample) were collected and centrifuged after being twice washed with phosphate-buffered saline (PBS). Each cell pellet was extracted by solvent composed of ice-cold chloroform (100 μl), methanol (200 μl), and 1 mg/ml of butylhydroxytoluene. After that, the samples were sonicated for 5 min (30-s sonication and 30-s rest cycle) on ice with a VCX 130 ultrasonicator. The samples were incubated for 30 min on ice and then centrifuged at 16,000 g for 20 min before being transferred into glass vials for liquid chromatography-mass spectrometry analysis. The supernatant was injected into a Waters Acquity UPLC CSH C18 column (100 × 2.1 mm; 1.7 μm) coupled to an Acquity UPLC CSH C18 VanGuard precolumn (5 × 2.1 mm; 1.7 μm). Lipidomics was run with an injection volume of 5 μl. Lipids were separated by gradient elution with solvents A (acetonitrile/water, 60:40, v/v) and B (isopropanol/acetonitrile, 90:10, v/v) both containing 10 mM ammonium acetate. Separation was performed at 65 °C with a flow rate of 0.6 ml min^−1^, conducted under the following gradient: 0 to 2 min, 15 to 30% B; 2 to 2.5 min, 30 to 48% B; 2.5 to 11 min, 48 to 82% B; 11 to 11.5 min, 99% B; 11.5 to 12 min, 99% B; 12 to 12.1 min, 99 to 15% B; followed by 4-min postrun at 15% B. Mass spectrometry analysis was performed on Thermo Scientific Q Exactive Plus Quadrupole-Orbitrap (Thermo Fisher Scientific) equipped with a heated electrospray source and operated in negative ion mode with the following parameters: sheath gas, 60 arbitrary units; auxiliary gas, 25 arbitrary units; sweep gas, 2 arbitrary units; spray voltage, 3 kV; capillary temperature, 320 °C; S-lens RF level, 50%; and aux gas heater temperature, 370 °C. Data-dependent acquisition (DDA) mode analysis was performed for quantification at MS1 mass range 120 to 1,200, resolution 70,000 at mass/charge ratio (*m*/*z*) 200, automatic gain control target of 1 × 10^6^, and a maximum injection time of 100 ms. For MS/MS acquisition, the parameters were as follows: resolution, 17,500 at *m*/*z* 200; automatic gain control target 1, × 10^5^; maximum injection time of 50 ms; Top N 4, isolation window, *m*/*z* 1.0; normalized stepped collision energy, 10-20-30. Using MS-DIAL software, lipid peaks were identified, aligned, and exported. The analysis only included lipids that could be fully identified by MS2 spectra.

### Statistical analysis

Graphical data are presented as the mean ± standard error of the mean (SEM). Statistical significance for more than 3 groups was determined using 1-way or 2-way analysis of variance (ANOVA) followed by a Tukey multiple comparison posttest. Statistical significance between 2 groups was analyzed using unpaired Student *t* tests. Statistical analysis was performed with GraphPad Prism 8 software. Significance was assumed for *P* < 0.05 (*), *P* < 0.01 (**), *P* < 0.001 (***), and *P* < 0.0001 (****).

## Results

### Elevated glycolysis in metabolic altered hESCs

**Fig. 1.**
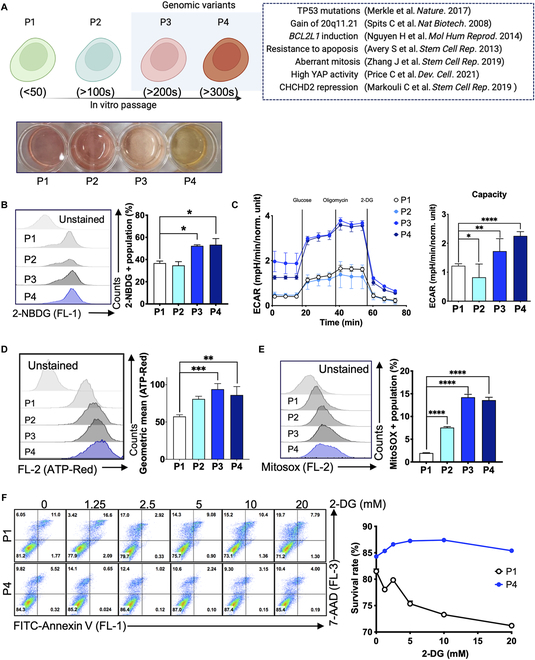
Elevated glycolysis in metabolic altered hESCs. (A) The graphical scheme of the isogenic hESC model with indicated passage numbers (P1: <50, P2:>100s, P3:>200s, and P4:>300s), used in this study (left). The phenotypes previously observed in the genomic variants (i.e., P3 and P4) and the corresponding reference reported from other groups (right, dotted box). The images of cell plates of P1, P2, P3, and P4 (bottom). (B) The level of glucose uptake in the isogenic pair was detected by 2-(N-(7-nitrobenz-2-oxa-1,3-diazol-4-yl)amino)-2-deoxyglucose (2-NBDG) staining. 2-NBDG was treated for 2 h, and then data was collected by flow cytometry after 3 times of PBS wash. The amount of the group with a high 2-NBDG ratio was regarded as glucose intake, quantified, and displayed in the right panel. (*n* = 3 independent experiments; mean ± SEM, 1-way ANOVA, *P < 0.05) (C) Glycolysis activity was measured by ECAR analysis as described in the method. Glucose capacity was determined by subtraction of nonglycolytic acidification from the highest ECAR. (*n* = 5 independent experiments; mean ± SEM, 1-way ANOVA, *P < 0.05, **P < 0.001, and ***P < 0.001) (D) ATP synthesis rate in the isogenic pair P1-P4 were detected by ATP-Red1 staining. The cells were treated with 1 μM ATP-Red for 1 h on the ordinary media (StemMACS), and then data was collected by flow cytometry after 3 times of PBS wash. The geometric mean, analyzed by FlowJo following the manufacturer’s manual was presented in the right panel. (*n* = 3 independent experiments; mean ± SEM, 1-way ANOVA, **P < 0.01, and ***P < 0.001) (E) The mitochondrial ROS production was examined by 10 μM MitoSOX staining for 1 h by flow cytometer analysis. (*n* = 3 independent experiments; mean ± SEM, 1-way ANOVA, ****P < 0.0001) (F) Cell death of Normal (P1) and Variant (P4) after 24 h of 2-DG treatment was analyzed as described in the method.

An isogenic set of H9 hESCs, spanning various passages (up to 300 passages), has been employed to characterize the biological consequences of common genetic changes that frequently occur in hPSCs during in vitro cultivation [10,12,28,29]. These changes, observed in “culture-adapted” hESCs, include TP53 mutations [4], copy number variation of 20q11.21 [5], repression of *CHCHD2* [30] induction of *BCL2L1* [8], aberrant mitosis [31], and high YAP activity [11] (Fig. 1A, right panel). Here, we observed that P3 (over 200 passages) and P4 (over 300 passages) hESCs, which exhibit distinct “culture-adapted phenotypes”, displayed a noticeable trend of medium acidification (Fig. 1A, bottom panel), although their cell growth rates were significantly slower than those of P1 and P2 hESCs (Fig. S1A and B). The absence of mycoplasma contamination in hESC culture (Fig. S1C) implies that the distinct metabolic changes occurred in these genetic variants, as expected. Given the pronounced reliance of primed hESCs on glycolysis [14], this increased acidification likely results from excessive glycolytic activity in these genetic variants.

Notably, the genetic variants also exhibit heightened glucose uptake (Fig. 1B), accompanied by an up-regulation of *SLC2A1* (which encodes glucose transporter 1) (Fig. S1D) and a substantial increase in extracellular acidification rate (ECAR) profiles (Fig. 1C). Therefore, these metabolic alterations constitute a key aspect of the “culture-adapted phenotypes”. Although the alteration in the oxygen consumption rate in P4 hESCs (Fig. S1E) was less noticeable compared to ECAR changes (Fig. 1C), elevated levels of total intracellular ATP were detected, as determined by ATP-Red1 (Fig. 1D), and mitochondrial reactive oxygen species (ROS) (Fig. 1E) in these genetic variants compared to normal hESCs.

Considering the distinct glucose metabolism of these genetic variants (P3 and P4 hESCs) compared to normal hESCs (P1 and P2 hESCs), we categorize these variants as “metabolic variants” (hereinafter referred to as “variants” for P3 and P4 hESCs versus “normal” for P1 and P2 hESCs). In light of the potential risks associated with abnormal variants in hESC-based stem cell therapy [32], selectively eliminating these variants constitutes a promising approach to ensure safe clinical applications. Therefore, we hypothesized that the enhanced glycolysis, as indicated by high glucose uptake and increased oxygen consumption rate, would make the variants more sensitive to glycolysis inhibition. However, it appears that P4 hESCs were notably resistant to glycolysis inhibition compared to P1 hESCs (Fig. 1F).

### High intracellular polyunsaturated lipid accumulation in metabolic variant hESCs

**Fig. 2.**
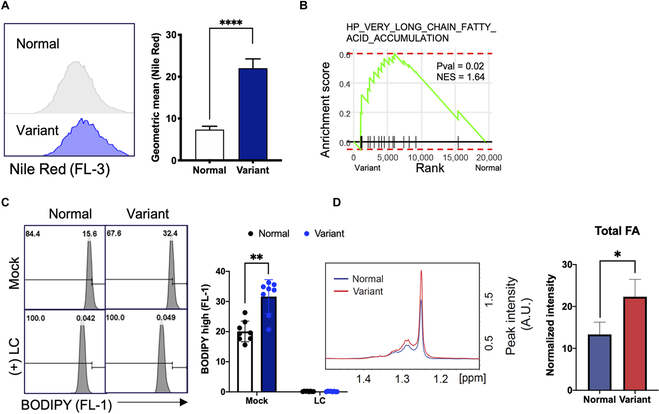
High intracellular polyunsaturated lipid accumulation in metabolic variant hESCs. (A) Lipid contents in Normal hESC and metabolic variant hESCs were quantified by Nile Red staining. The dye was added to the media by 1:1,000 concentration for 1 h and then analyzed and quantified by a flow cytometer. The right panel indicates geometric mean of Nile Red staining. (*n* = 4 independent experiments; mean ± SEM, unpaired *t* test, ****P < 0.0001) (B) GSEA analysis of Variant versus normal based on microarray data from Gene Expression Omnibus. (C) The amount of lipid in Normal and Variant cell was quantified by 1:1,000 BODIPY staining with or without 24 h of LC treatment. The right panel shows the quantification of geometric mean of BODIPY. The data was collected and quantified as described. (*n* = 4 independent experiments; mean ± SEM, unpaired *t* test, **P < 0.01) (D) NMR quantification of total fatty acid (FA) in Normal and Variant. (Left) The overlaid 1D proton spectrum for Normal (blue) and Variant (red) cells in the region of total FAs (1.2 to 1.4 ppm). The representative spectrum for each cell is shown. (Right) Comparison of amount of total FAs between Normal and Variant cells. (*n* = 3 independent experiments; mean ± SEM, unpaired *t* test, *P < 0.05).

In contrast to somatic cells, hPSCs display a distinct preference for utilizing fatty acids as their primary energy source to withstand metabolic stress. This reliance on fatty acids is attributed to their essential role in both sustaining survival [18] and maintaining pluripotency [17]. To support this, we conducted an analysis of DEGs between undifferentiated hPSCs and their differentiated counterparts (Fig. S2A). Notably, gene sets associated with “fatty acid biosynthesis” and “fatty acid metabolism” were markedly enriched in undifferentiated hPSCs (Fig. S2B). In light of these findings, it was intriguing to observe a substantial intracellular fatty acid level in the variants (Fig. 2A), which corresponded with the enrichment of gene sets related to “fatty acid accumulation” (Fig. 2B). Furthermore, the marked reduction of intracellular fatty acids following L-carnitine (LC) treatment indicated active FAO in both normal and variant hESCs (Fig. 2C). As depicted in Fig. S2C, the higher level of total fatty acids, determined by NMR, indicates enhanced lipogenesis (Fig. 2D), accompanied by high lactate production in the variants (Fig. S2D). Interestingly, LC treatment in the variants attenuated their resistance to glycolysis inhibition (Fig. S2E). The result indicates the fatty acid consumption was able to mask the effect of decreased glycolysis, suggesting that the elevated fatty acid storage in the variants serves as an alternative energy source in addition to glucose.

### High ferroptosis susceptibility of the metabolic variants

**Fig. 3.**
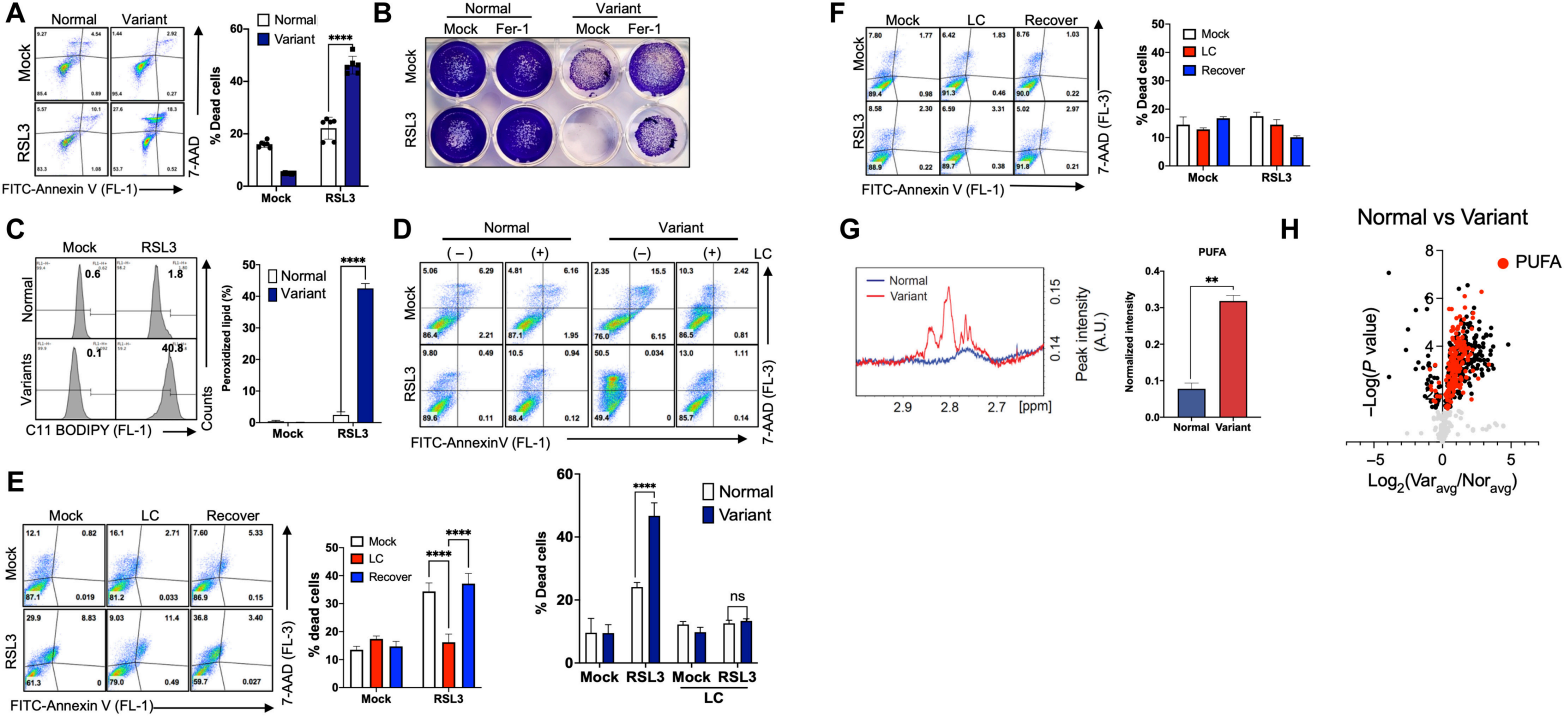
High ferroptosis susceptibility of the metabolic variants. (A) The effect of 500 nM RSL3 for 24 h treatment on Normal and Variant was tested by 7-AAD and Annexin V staining. The right panel indicates cell death, analyzed and quantified as described. (*n* = 3 independent experiments; mean ± SEM, unpaired *t* test, ****P < 0.0001) (B) Cellular growth of Normal and Variant under 500 nM RSL3 with or without 500 nM of Fer-1 treatment were examined by clonogenic assay. The ferrostatin was pretreated 24 h before RSL3 treatment, and RSL3 and ferrostatin were cotreated for extra 24 h. (C and D) The quantification on the right panel was collected and analyzed by fluorescence-activated cell sorting (FACS) and FlowJo as described. (C) Lipid peroxidation was tested and quantified by BODIPY 581/591 C11 (C11 BODIPY) staining after 4 h of RSL3 500 nM treatment. (*n* = 3 independent experiments; mean ± SEM, unpaired *t* test, ****P < 0.0001) (D) The amount of cell death of Normal and Variant hESCs under 500 nM RSL3 treatment with, or without 24-h pretreatment of LC. LC was included in the media during RSL3 treatment. (*n* = 3 independent experiments; mean ± SEM, unpaired *t* test, ****P < 0.001) (E and F) The cell death under 500 nM of RSL3 was tested. LC was pretreated for 24 h in LC and recover sample and then removed only in the recover sample during 24 h of RSL3 treatment. (E) Experiments for Variant hESCs. *n* = 3 independent experiments; mean ± SEM, 2-way ANOVA, ****P < 0.001. (F) Experiments for Normal hESCs. (G) The overlaid 1D proton spectrum for Normal (blue) and Variant (red) cells in the region of PUFAs (2.7 to 2.9 ppm). The representative spectrum for each cell is shown. (Right) Comparison of the amount of PUFAs between Normal and Variant cells. A.U., arbitrary unit. (H) Volcano plot illustrating lipidomic features in normal and variant stem cells. Each data point represents an individual lipid species. Lipids containing PUFAs are highlighted in red.

The most well-characterized feature of genetic variant hPSCs is their survival trait, particularly their resistance to stress-induced apoptosis [8]. This resilience is consistently observed, even when challenged with compounds such as YM155 [33], a small molecule known to selectively induce apoptosis in hPSCs through the SLC35F2 transporter [34], or nocodazole (Noc) [10] (Fig. S3A and B). Given the strong resistance of the variants to apoptosis, we further challenged them with ferroptosis, another form of programmed cell death, is closely linked to metabolism and ROS [35]. Contrary to our expectations, the genetic variants displayed heightened sensitivity to RSL3 (i.e., a typical ferroptosis inducer by GPX4 inhibition) compared to normal hESCs (Fig. 3A). This cell death was reversed through treatment with the ferroptosis inhibitor, ferrostatin-1 (Fer-1), suggesting that the cell death observed in the variants was attributed to ferroptosis (Fig. 3B). Notably, the level of peroxidized lipids dramatically increased following RSL3 treatment, specifically in the variants (Fig. 3C). Given the elevated levels of total fatty acids, including both free and lipid-contained forms, in the variants (Fig. 2E), it appears that these fatty acids contribute to the susceptibility to ferroptosis. To test this hypothesis, we utilized LC, which promotes FAO and reduces fatty acid levels, and then assessed the sensitivity of the variants to RSL3. As expected, the use of LC significantly reduced the sensitivity to RSL3 (Fig. 3D). Interestingly, upon the removal of LC, intracellular total lipid levels rapidly increased in the variants (Fig. S3C), leading to cell death upon RSL3 treatment (Fig. 3E), while no clear cell death was observed in normal hESCs (Fig. 3F). NMR results indicate that the levels of total UFAs, especially PUFAs, were markedly increased in the variants compared to normal hESCs (Fig. 3G and Fig. S3D). The consequent lipidomic analysis also confirmed the increased PUFA-containing phospholipids (PUFA-PLs) in the variants (Fig. 3H and Table S1). Notably, PUFAs are prone to peroxidation, and this peroxidation process, characterized by a chain reaction, leads to ferroptosis [36]. Therefore, we speculated that high PUFA incorporation in the variants may account for the ferroptosis vulnerability. However, the introduction of fatty acids into normal hESCs through Albumax supplementation (a lipid-rich bovine albumin serum) (Fig. S3E) gave only marginal effect on their susceptibility to ferroptosis (Fig. S3F). These results suggest that a type of molecular event, specifically occurring in the variants, would be necessary for ferroptosis induction.

In order to rule out the possibility that this unexpected ferroptosis susceptibility in H9 hESCs (this model) occurs in a cell-type-specific manner, the heightened susceptibility to ferroptosis observed in the variants was reproduced in iPSCs with trisomy at chromosome 12 (iPSC-T12), one of the recurrent genetic variations in hPSCs [6]. The iPSC-T12 with the robust survival trait due to high BCL2L1 expression [37], displayed elevated accumulation of total fatty acids (Fig. S4A) and increased susceptibility to ferroptosis induced by RSL3 (Fig. S4B and C), which was inhibited by Fer-1 (but not Z-VAD) (Fig. S4D), along with lipid peroxidation (Fig. S4E). Similar to the variants from hESCs (Fig. 3F), fatty acid expenditure by LC treatment rescued ferroptosis induction in iPSC-T12 (Fig. S4F).

### YAP-dependent induction of ASCL4 in the variants for ferroptosis susceptibility

**Fig. 4.**
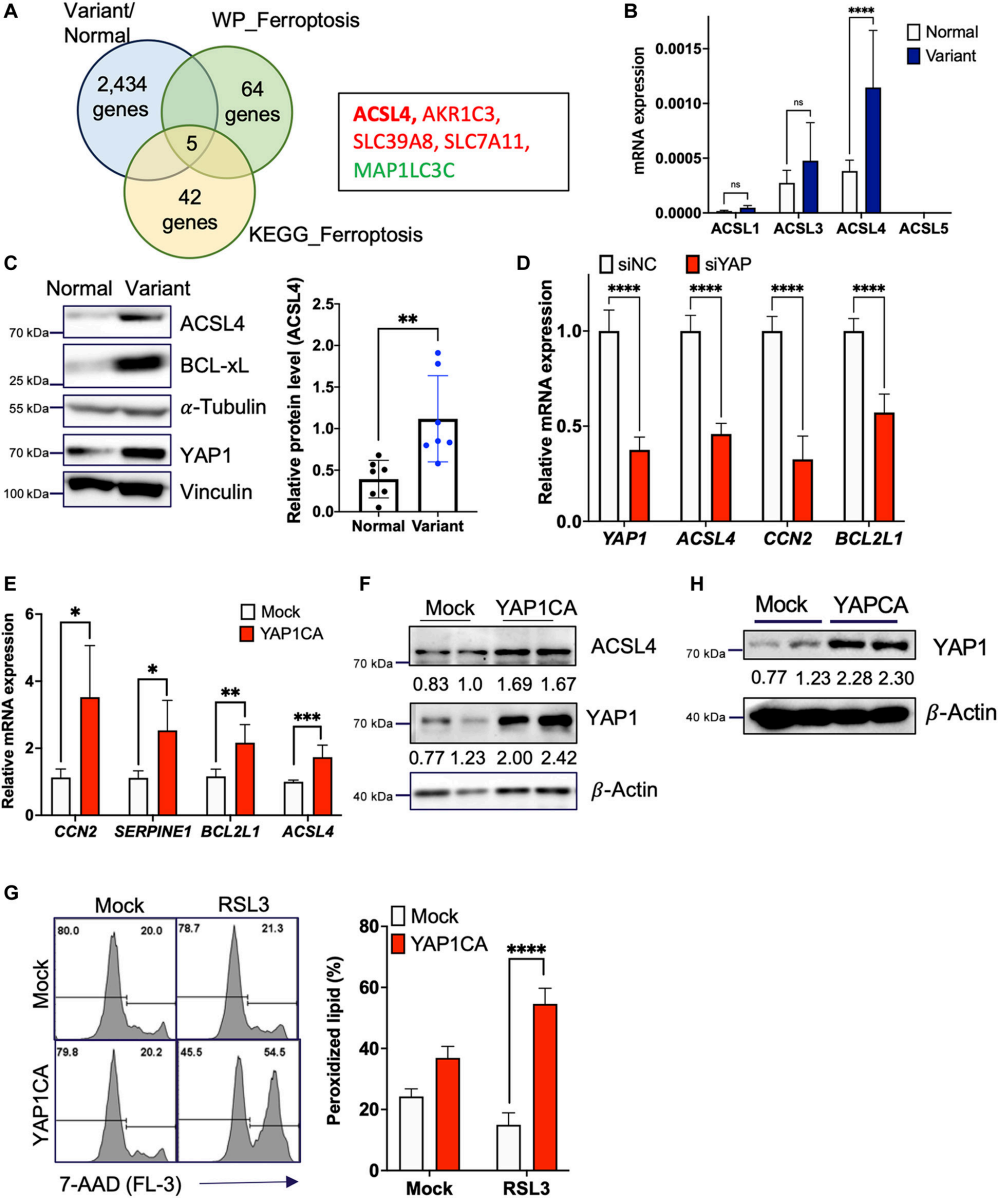
YAP-dependent induction of ASCL4 in the variants for ferroptosis susceptibility. (A) Venn diagram for commonly altered genes from the variants and 2 typical ferroptosis gene sets (left panel), up-regulated genes: red and down-regulated gene: green in the box (right panel). (B) Relative mRNA expression level of ACSL1, 3, 4, and 5. (C) Protein expression level of ACSL4. BCL-xL is a positive control that indicates Variant hESCs. *α*-tubulin as loading control. Right panel represents the quantification of ACSL4 Immunoblotting intensity (normalized by *β*-actin intensity). *n* = 7, paired *t* test, **P < 0.01 (D) High YAP1 and ACSL4 levels were indicated in Variant hESC compared to normal. Vinculin is a housekeeping gene, considered as a loading control. (E and F) Housekeeping gene 18S ribosomal RNA and glyceraldehyde-3-phosphate dehydrogenase were used for normalization. (E) The mRNA level of ACSL4 and YAP1 downstream genes were analyzed in the YAP1 transient knockdown model. (*n* = 3 independent experiments; mean ± SEM, unpaired *t* test, ****P < 0.0001) CA, continuously active form. (E) *CCN2*, *SERPINE1*, and *BCL2L1*, the downstream gene of YAP1, and ACSL4 expression were tested in the YAP18SA overexpression model. (*n* = 3 independent experiments; mean ± SEM, unpaired *t* test, *P < 0.05, **P < 0.01, ***P < 0.001) (F) The protein level of ACSL4 was tested in the YAP18SA overexpression model. *β*-Actin as a loading control. (G) The YAP1 protein level after overexpression were tested in 2 samples used in cell death analysis. *β*-Actin as loading control. The band intensity of immunoblotting data for (B), (C), (F), and (H) was shown below. (H) Cell death under 500 nM RSL3 treatment was analyzed by FACS after 7-AAD staining. The right panel shows the quantification result of cell death. (*n* = 3 independent experiments; mean ± SEM, unpaired *t* test, ****P < 0.001). KEGG, Kyoto Encyclopedia of Genes and Genomes.

The molecular mechanism underlying the selective vulnerability to ferroptosis was examined in more detail by analyzing the DEGs from the variants (i.e., P3 and P4) compared to normal hESCs (i.e., P1 and P2 hESCs) using data from BioProject: PRJNA1020991. As shown in Fig. 4A, out of a total of 64 genes from the WP_ferroptosis pathway (https://www.wikipathways.org/pathways/WP4313.html) and 42 genes from the Kyoto Encyclopedia of Genes and Genomes ferroptosis pathway (https://www.genome.jp/entry/pathway+hsa04216 and Fig. S5A), 5 genes (4 up-regulated and 1 down-regulated) were identified in the variants (Fig. 4A, left panel). Unlike NRF2 downstream genes such as AKR1C3 and SLC7A11, which typically repress ferroptosis [38], the induction of ACSL4 was evident, indicating its role in the susceptibility to ferroptosis, as previously described [39]. Notably, ACSL3, responsible for MUFA synthesis, repressing ferroptosis [40] remained unaltered in the variant (Fig. 4B). The protein expression of ACSL4 was clearly up-regulated in the variants, along with BCL-xL, a typical marker for the variants (Fig. 4C). The distinct resistance to apoptosis in the variants, which provides a competitive advantage over normal cells, results from increased YAP1 activity [11] or YAP1-dependent *BCL2L1* expression [10]. However, unlike the clear survival advantage against apoptosis, YAP1 activity promotes ferroptosis by directly inducing ACSL4 expression in a TEAD4-dependent manner [41,42].

Therefore, we speculated that the high expression of ACSL4 in the variants, in parallel with high YAP1 protein levels (Fig. 4C) and high TEAD4 protein levels (Fig. S5B), would be responsible for the high RSL3 sensitivity. Consistent with previous studies [41], the depletion of YAP1, which led to the suppression of typical YAP1 downstream genes (e.g., *CCN2* and *BCL2L1*), attenuated *ACSL4* expression (Fig. 4D). Conversely, the ectopic expression of constitutively active YAP1 (i.e., Flag-YAP1-8SA mutant: YAP1CA) in normal hESCs (Fig. S5C) enhanced ACSL4 transcription but not ACSL3 (Fig. S5C), along with typical YAP1 downstream genes such as *CCN2*, *SERPINE1*, and *BCL2L1* (Fig. 4E) as well as ACSL4 protein level (Fig. 4F). This ectopic expression of YAP1 in normal hESCs (Fig. 4H) significantly increased their sensitivity to RSL3 (Fig. 4G). Additionally, the YAP1 activation and the ACSL4 induction were recapitulated in iPSC-T12, a ferroptosis-sensitive iPSC variant, as shown in Fig. S4 (Fig. S5D). These findings indicate that YAP1-dependent *ACSL4* expression is critical for the ferroptosis vulnerability in the variants of hPSCs.

### ACSL4 determines ferroptosis vulnerability in the variant hESCs

**Fig. 5.**
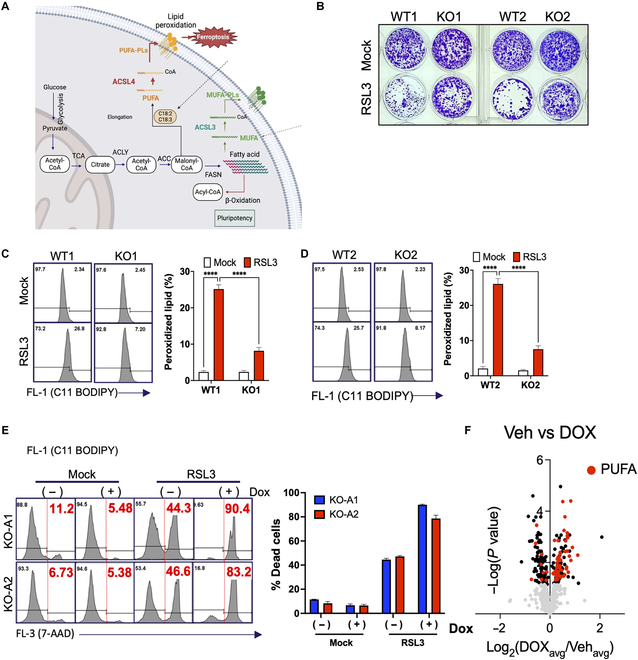
ACSL4 determines ferroptosis vulnerability in the variant hESCs. (A) Graphical summary of fatty acid synthesis from glucose uptake and role of ACSL families in ferroptosis. (B) Sensitivity of ACSL4 KO-Variant cell under 500 nM of RSL3 treatment compared to wild type (WT). Each number indicates different clones. (C and D) Lipid peroxidization of WT and KO cells after 3 h of 1,000 nM RSL3 treatment were tested by C11 BODIPY staining. (C) KO and WT clone set 1 and (D) KO and WT clone set 2 (*n* = 3 independent experiments for each figure; mean ± SEM, 2-way ANOVA, ****P < 0.001). (E) Cell death of Dox-inducible ACSL4 reconstruction model clone 1 (KO-A1) and clone2 (KO-A2) under 3,000 nM of RSL3 treatment for 24 h. Cells were treated with Dox (1 mg/ml) for 72 h, including 48 h of pretreatment and 24 h of RSL3 cotreatment. (F) Volcano plot of lipidomic features in ACSL4-KO and Dox-induced ACSL4-restored cell line. Each data point represents an individual lipid species. The lipids containing PUFAs are highlighted in red.

ACSL4 plays a crucial role in converting PUFAs into acyl-CoA (PUFA-CoA), ultimately leading to an increase in PUFA-PLs). The oxidation of PUFA-PLs triggers a chain reaction of lipid peroxidation, which in turn causes ferroptosis [43]. In contrast, ACSL3 produces monounsaturated fatty acyl-CoA (MUFA-CoA) to form MUFA-containing phospholipids (MUFA-PLs) to confer ferroptosis resistance [40] (Fig. 5A). For a more detailed investigation of ACSL4 in the variants, ACSL4 KO was carried out using CRISPR/Cas9 to generate 2 independent clones (KO1 and KO2) with different insertion and deletion (indel) mutations (Fig. S6A). Alkaline phosphatase activity and the level of *POU5F1* (Fig. S6B) revealed that the loss of *ACSL4* had a marginal effect on the pluripotency of hESCs. The loss of ACSL4 protein (Fig. S6C) clearly rescued RSL3-induced cell death (Fig. 5B) and significantly reduced the level of lipid peroxidation caused by RSL3 treatment (Fig. 5C and D). The requirement of ACSL4 expression for ferroptosis susceptibility was highlighted by the doxycycline (Dox)-inducible re-expression of ACSL4 (Fig. S6D). The tet-ON inducible *ACSL4*, stably expressed in KO (KO-A1 and KO-A2), successfully expressed ACSL4 with simple Dox treatment (Fig. S6E). Dox-induced ACSL4 expression markedly sensitized KO cells to RSL3-induced cell death (Fig. 5E), which was completely inhibited by Fer-1 treatment (Fig. S6F). Additionally, lipidomics analysis revealed that the overall level of PUFA-PLs increased after the re-expression of ACSL4 in the KO variants (Fig. 5F and Table S2).

### Selective elimination of the variants via brief RSL3 treatment

**Fig. 6.**
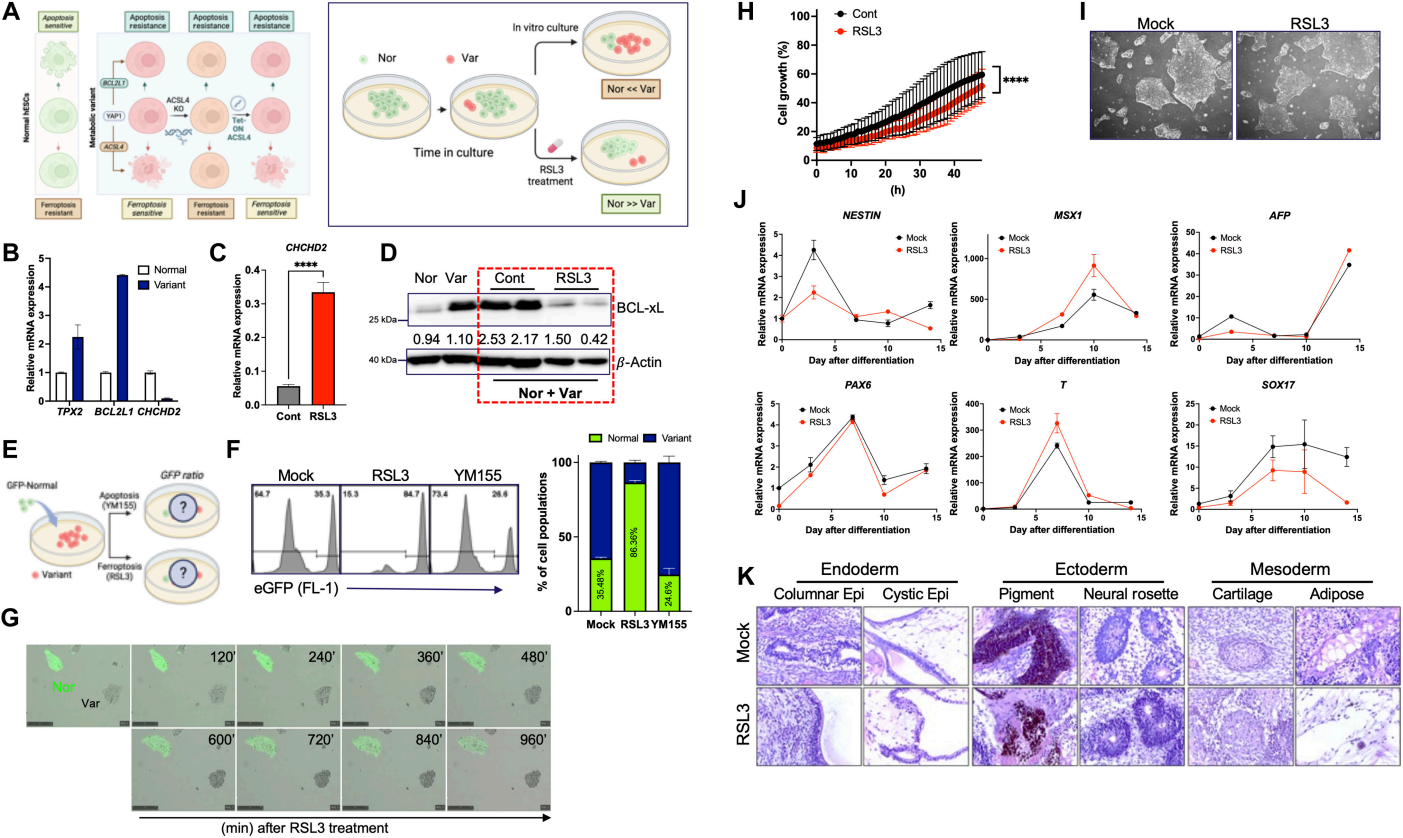
Selective elimination of the variants via brief RSL3 treatment. (A) Representative figures of selective elimination hypothesis. (B) mRNA expression level of Genetic alteration marker in Normal and Variant hESCs. (C and D) The normal and variant cells were 1:1 mixed and treated for 48 h with 250 nM RSL3. (C) CHCHD2 mRNA expression level (*n* = 2 independent experiments; mean ± SEM, unpaired *t* test, ****P < 0.0001) and (D) BCL-xL protein expression level was tested. *β*-Actin as loading control. (E) Graphical summary of suggested reaction of Normal cell tagged with enhanced GFP and Variant cells under RSL3 and YM155 treatment. (F) The normal cell population was quantified by enhanced GFP level by FACS after treatment of 250 nM RSL3 for 48 h and 20 nM of YM155 for 24 h. The right panel indicates the quantification result of the population (%). (G) Representative pictures show that Normal (Nor) and Variant (Var) cells respond differently to 250 nM RSL3 treatment per 16 h on the same plate. (H) Growth curve of Normal hESCs (H9) under RSL3 treatment. The live cell imaging and analysis were done as described. (*n* = 3 independent experiments for each figure; mean ± SEM, 2-way ANOVA, ****P < 0.001). (I) Bright-field image of normal hESCs (H9) after 250 nM RSL3 exposure for 48 h. (J) Three-germ-layer-specific marker gene expression was analyzed through reverse-transcription quantitative polymerase chain reaction after spontaneous differentiation of Mock and RSL3-treated Normal hESCs (MSX1 and T for mesoderm, PAX6 and NESTIN for ectoderm, and SOX17 and AFP for endoderm marker genes; *n* = 2 independent experiments; mean ± SEM). (K) In vivo 3-germ-layer differentiation of Mock and RSL3-treated H9 cells was tested. The teratoma was collected and sectioned and then stained by hematoxylin and eosin. The following were shown: Endoderm structure: columnar epithelium (Epi) and cystic epi. Ectoderm structure: Pigment and neural rosette. Mesoderm structure: Cartilage and adipose tissue (Adipose).

While these variants exhibit high resistance to apoptosis due to YAP1-dependent *BCL2L1* induction, paradoxically, these cells display clear vulnerability to ferroptosis as a result of YAP1-dependent ACSL4 expression (Fig. 6A, left panel). Such distinct characteristics of the variants, namely ferroptosis vulnerability, can be utilized to counter their competitive advantage over normal hESCs during in vitro culture [11]. Therefore, a transient exposure to RSL3, which has a marginal effect on normal hESCs, can be applied for the selective elimination of the variants in culture (Fig. 6A, right panel). To test this idea, variant hESCs (Var) were cocultured with normal hESCs (Nor) to mimic the in vitro competition between normal and variant hESCs. Along with *TPX2* and *BCL2L1*, which are highly expressed in the variants [10,12] (Fig. 6B), the distinct repression of coiled-coil-helix-coiled-coil-helix domain containing 2 (CHCHD2) was evident, as described previously (Fig. 6C) [28]. CHCHD2 serves as an effective indicator of the competition’s outcome. The level of CHCHD2 in the cell mixture of normal and variants was noticeably increased after 48 h of RSL3 treatment (Fig. 6C). Similarly, the high BCL-xL protein level (a typical marker for the variants) in the mixture was also attenuated by RSL3 treatment (Fig. 6D). To demonstrate that the alteration in the levels of CHCHD2 (Fig. 6C) and BCL-xL (Fig. 6D) results from the selective elimination of the variants by RSL3, green fluorescent protein (GFP)-expressing normal hESCs were cocultured with the variants, followed by treatment with either YM155 (for apoptosis induction) or RSL3 (for ferroptosis induction) (Fig. 6E). The GFP-positive normal hESCs, which lost the competition under apoptosis conditions with YM155 (Fig. S7A), appeared to outcompete the variants under ferroptosis conditions with RSL3 (Fig. 6F and G). Selective ferroptotic cell death in the variants, compared to normal hESCs (GFP+), was clearly observed in live images following RSL3 treatment (Movie S1B). This contrasts with the comparable growth observed under standard culture conditions (Movie S1A) and the marked resistance to apoptotic cell death induced by YM155 (Movie S1C). The distinct responses to ferroptosis (induced by RSL3) and apoptosis (induced by YM155) between normal and variant hESCs are further highlighted in Fig. S7B.

To apply this approach for enriching normal hESCs, it is essential that short-term RSL3 treatment does not impair the characteristics of hESCs. Despite moderate growth retardation caused by RSL3 treatment (Fig. 6H), normal hESCs exposed to RSL3 retained their typical characteristics, such as cell morphology (Fig. 6I), alkaline phosphatase activity (Fig. S7C), and marker expression (Fig. S7D). Moreover, the maintenance of cellular features in normal hESCs after exposure to RSL3 was highlighted through the observations of spontaneous differentiation (Fig. 6J and Fig. S7E) and the formation of teratomas exhibiting distinct 3 germ layers (Fig. 6K and Fig. S7F). Importantly, these outcomes closely resembled those of the control hESCs that were not exposed to RSL3. These findings provide clear evidence that a brief course of RSL3 treatment can electively eliminate the variants while minimally affecting coexisting normal hESCs.

## Discussion

Our uniquely designed isogenic hESCs models, featuring exaggerated characteristics, effectively replicate the broad spectrum of biological responses observed in culture-adapted hPSCs. These culture-adapted phenotypes are clearly associated to the recurrent genetic alterations, a phenomenon consistently documented across numerous independent studies [10,12] (as illustrated in Fig. 1A). Therefore, these cells have become a unique model, enabling the study of severe consequences resulting from genetic anomalies, such as the gain of 20q11.21 and/or TP53 mutations [29]. In addition to the survival traits stemming from *BCL2L1* induction [8], YAP1 activity [11], the decoupling of Rho A–ROCK signaling [44], and abnormal mitosis [12], this study revealed a distinct increase in glycolytic dependency, a hallmark of primed ESCs [14]. This metabolic shift resulted in an elevated production of fatty acids, representing a novel “culture-adapted phenotype”. Given the pivotal roles of UFAs in pluripotency maintenance [23], the effects of MUFAs and PUFAs on ESCs are evidently different. Particularly, the augmented levels of MUFA, associated with acquired pluripotency [24], play a crucial for the survival of hESCs [25]. Conversely, the inhibition of fatty acid polydesaturation rescues ESCs from cell death while preserving pluripotency [26]. These studies suggest that MUFA, as opposed to PUFA, is beneficial for the survival and pluripotency of ESCs.

In this study, we demonstrated that drastic metabolic changes such as increased glycolysis (Fig. 1) and fatty acid synthesis (Fig. 2) were accompanied with the elevated level of PUFA in the variants (Fig. 3). This alteration resulted in a pronounced dependency on GPX4 activity (Fig. 3). The uncertainty surrounding the altered biological characteristics resulting from genetic alterations in cultured hPSCs remains a serious concern for the safety of the clinical application of therapeutic cells derived from hPSCs [7,32]. Multiple studies have even demonstrated the formation of somatic tumors after transplanting differentiated cells derived from aberrant hPSCs [45]. In particular, these variants outcompete normal hPSCs by acquired survival trait, the most extensively characterized “culture adapted phenotype” and consistently dominate during repeated in vitro culture [11]. Consequently, addressing critical challenges for the clinical use of hPSCs has earned increasing interest, including the identification of biomarkers for selective isolation [30], the development of culture condition to minimize such occurrences [46], and the induction of selective cell death of the variants [6]. In this regard, the unexpected susceptibility of “culture-adapted hPSCs” to ferroptosis (Fig. 3 and Fig. S3) presents a promising avenue for eliminating the aberrant hPSCs through straightforward treatment with GPX4 inhibitors (such as RSL3) (Fig. 6). The production of MUFAs, which is crucial for the survival of hPSCs [25], inhibit the peroxidation of PUFA-PLs [40]. In normal hPSCs, this MUFA production likely contributes to the resistance to ferroptosis in contrast to variant hPSCs, which have elevated PUFA production. Apart from one study that linked dissociation-induced cell death in hESCs to ferroptosis [47], no other research has been reported on ferroptosis in hPSCs, suggesting the inherent resistance of normal hPSCs to ferroptotic cell death.

Our findings revealed that YAP1-dependent ACSL4 expression, elevating the levels of PUFA-PLs [48], was responsible for ferroptosis susceptibility in the variants from hESCs (Fig. 4) and iPSCs (Fig. S4). Importantly, high YAP1 activity, outcompeting normal hPSCs [11] through inducing *BCL2L1* [10], ironically engender ferroptosis susceptibility through ACSL4 expression in the variant. Thereby, the selective elimination of “hPSCs with high YAP1 activity” (i.e., the culture adapted hPSCs) through iGPX4 would be likely achieved in other hPSC models.

Under the chemically defined culture conditions that favor lipogenesis, the metabolic alterations in the variants resulted in the accumulation of PUFA compared to normal hESCs (Fig. 3G and H). Interestingly, elongation of very long-chain fatty acid protein 5 (*ELOVL5*), one of the PUFA elongases [49], was highly expressed in the variants. Although the KO of *ELOVL5* only partially rescued RSL3-mediated ferroptosis in the variants (data not shown), this implies that the PUFA-PLs produced from the incorporated PUFA by ACSL4 expression were sufficient to render the variants vulnerable to ferroptosis. Although we unveiled that metabolic alteration in the variant was accompanied with the elevated PUFA, it remains unclear how PUFA accumulation occurs in the variants. This intriguing question warrants further investigation in future studies.

In conclusion, the unique susceptibility to ferroptosis, driven by elevated PUFA levels and YAP1-dependent ACSL4 induction, would represent a novel “culture-adapted phenotype” in genetically aberrant hPSCs. Consequently, a straightforward application of iGPX4 presents an advantageous approach for selectively eliminating apoptosis-resistant “culture-adapted hPSCs” in in vitro culture. Given the minimal impact of short course iGPX4 treatment on normal hPSCs, regular application during long-term in vitro culture of hPSCs would be recommended to mitigate the emergence of the aberrant hPSCs’ dominance.

## Ethical Approval

This study using hESCs lines was approved by the Public Institutional Bioethics Committe designated by the Ministry of Health and Welfare (Seoul, Republic of Korea; IRB no. P01-201409-ES-01).

## Data Availability

Source data are available upon request. The original immunoblots were presented in Fig. S8.
