# Supplementary material for: Selective Elimination of Genetic Variants of Human Embryonic Stem Cells from High Vulnerability to Ferroptosis
Source: Biomater Res. 2024 Oct 25;28:0093. doi: 10.34133/bmr.0093 (PMC12237090; doi:10.34133/bmr.0093)
Supplement: Supplementary 1 — Figs. S1 to S8 Tables S1 and S2 Movie S1 [file bmr.0093.f1.zip › 240820 Supplement info_Ferroptosis hESCs.pdf]

Supplemental Figure legends

Figure S1 Metabolic alteration in the variant hESCs (A) Growth rate of P1, P2, P3 and P4 by clonogenic assay. $4X10^3$ Cells were seeded and cultured for 1 week. %Area of 3 biological repeats were represented in the right panel. **(B)** Cell growth rate of Normal (P1) and Variant (P4) determined by live imaging **(C)** PCR for mycoplasma contamination in hESCs culture. **(D)** mRNA expression level of BCL2L1 and SLC2A1 was quantified in P1, P2, P3 and P4. **(E)** Oxygen consumption rate (OCR) in P1 and P4

Figure S2 High fatty acid synthesis in the variant hESCs (A) List of datasets used for GSEA assay **(B)** Geneset enrichment analysis (GSEA) in hESCs compared to the differentiated counterparts from the indicated datasets **(C)** General landscape of glucose uptake and fatty acid synthesis. Red indicates the number of carbon (C) **(D)** The level of lactate production in normal (P1) and variant (P4) determined by NMR. **(E)** Cell growth rate of variant (P4) PSCs under glycolysis inhibition and acceleration of β-oxidation was tested by clonogenic assay. 2-DG and L-Carnitine treated for 1day, and cells were cultured for extra 3 days before analysis.

Figure S3 Distinct response of apoptosis and ferroptosis in the variant hESCs (A) Clonogenic assay, under 20nM of YM155 and 20uM of Nocodazole (Noc.) in normal (P1) and variant (P4) models **(B)** Cell death of normal (P1) and variant (P4) were tested under 20uM of Nocodazole. The data was collected and analyzed by Flowjo after 7-AAD and Annexin-V staining for 1hr. (n=4 independent experiments; mean ± SEM, 2-way ANOVA, ***$p < 0.001$) **(C)** The Lipid content of Variant (P4) PSCs after LC treatment and recover. (n=3 independent experiments; mean ± SEM, One-way ANOVA, **<0.01) **(D)**The overlaid 1D proton spectrum for normal (P1, blue) and variant (P4, red) cells in the region of total UFAs (2.7 – 2.9 ppm) is shown in the left. The representative spectrum for each cell was shown as a bar graph. **(E)** Total amount of lipid was tested in normal (P1) cell, after 24hrs of Albumax supplement. The right

panel shows FACS quantification of BODIPY intensity. **(F)** Flowcytometry analysis with 1000nM of RSL3 was treated for 24hrs with 1.6%(w/v) of Albumax. Albumax was 24hr pre-treated to normal (P1) before RSL3 treatment.

Figure S4 Ferroptosis susceptibility in iPSCs with trisomy at chromosome 12 (A) Total lipid content quantified by BODIPY staining and Flow cytometer analysis in Normal BJ-iPSCs (iPSC), and chromosome 12 trisomy BJ-iPSCs (iPSC-T12). **(B)** Cell death analysis in Normal iPSCs (iPSC) and variant iPSCs (iPSC-T12). Ferrostatin-1(Fer-1) 500nM or ZVAD 20nM were pre-treated for 24hrs, then co-treated with 500nM RSL3 for 24hrs. After treatment, iPSCs were cultured for an extra 24hrs before clonogenic assay. **(C)** Cell death after 24hrs of 500nM of RSL3 treatment were analyzed by FITC-AnnexinV and 7-AAD co-staining. **(D)** Cell death of iPSC-T12 under indicated dose of RSL3 treatment for 24hrs. **(E)** Lipid peroxidation of indicated iPSCs was quantified by flowcytometry with BODIPY-C11. **(F)** Flow cytometry for determining cell death with or without LC by RSL3. LC was pre-treated for 24hrs, then co-treated for 24hrs with RSL3 500nM.

Figure S5 High ACSL4 expression in the variant hESCs (A) Ferroptosis pathway in KEGG map from the inhouse RNA-seq (high in the variants: Red, low in the variants: Green) **(B)** ACSL4 and TEAD4 protein expression of normal and variant hESCs was determined by immunoblotting. β-actin as loading control. **(C)** mRNA expression of YAP1 and ACSL3 after YAP1CA expression in normal hESCs **(D)** Immunoblotting of YAP1, BCL-xL and ACSL4 expression of BJ-iPSC WT and iPSC-T12. β-actin as loading control.

Figure S6 Establishment of ACSL4 cell models (A) The sequences of KO1 (- 10 base pairs) and KO2 (-8 base pairs) hESCs compared to WT. **(B)** Alkaline phosphatase (AP) activity (top) and higher magnification of colony (bottom), mRNA expression level of *POU5F1*(Right). **(C)** ACSL4 protein expression in WT1, WT2, KO1 and KO2. Vinculin and β-actin as loading

control. **(D)** Scheme of the expected phenotype of ACSL4 TET-On reconstruction cell line. **(E)** ACSL4 protein expression of KO-A1 and KO-A2 with or without 1mg/ml of Doxycycline (Dox) treatment. Vinculin as a loading control. **(F)** Cell death of KO-A1 and KO-A2 under 2000nM of RSL3 with or without Fer-1 24hrs pretreatment were tested by clonogenic assay. 1mg/ml of Dox was previously treated 24hrs before Fer-1 treatment and remains in media during 48hrs of assay.

Figure S7 Characterization of normal hESCs after RSL3 exposure (A) Time dependent images of normal (P1-EGFP, Nor) and variant (P4, Var) hESCs after 20nM YM155 treatment. **(B)** AP staining of normal hESCs, with or without 250nM RSL3 48hrs exposure. **(C)** Fluorescence microscopic images of OCT4 in normal hESCs, with or without 48hrs of 250nM RSL3 treatment. **(D)** mRNA expression level of *LIN28* and *POU5F1* in undifferentiated normal hESCs (Undiff) and spontaneous differentiated counterparts after 14 days differentiation (Diff). **(E)** Teratoma formed from mouse testis by injection of vehicle (Mock) or 250nM of RSL3 treated hESCs compared control testis (Cont).

Figure S8 Uncut immunoblotting data presented in the figures

Movie S1 (A) Live images of normal (with green fluorescence protein) and variants after treatment of Mock **(B)** 20nM of RSL and **(C)** 10nM of YM155

Figure. S1

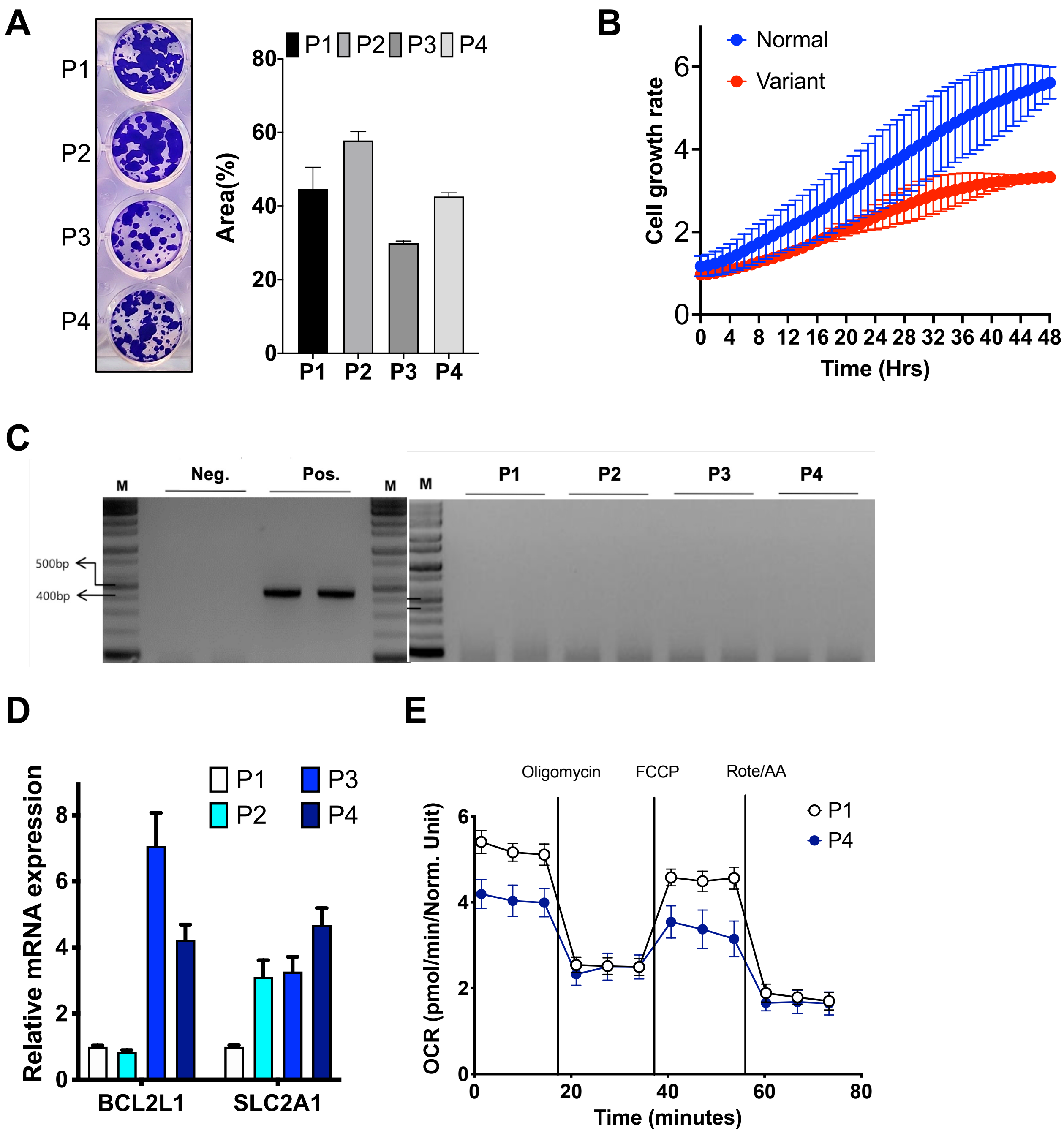
A
P1
P2
P3
P4
P1 P2 P3 P4
Area(%)
80
60
40
20
0
P1 P2 P3 P4
B
Normal
Variant
Cell growth rate
6
4
2
0
0 4 8 12 16 20 24 28 32 36 40 44 48
Time (Hrs)
C
M
Neg.
Pos.
M
M
P1
P2
P3
P4
500bp
400bp
D
Relative mRNA expression
8
6
4
2
0
P1 P3
P2 P4
BCL2L1
SLC2A1
E
Oligomycin
FCCP
Rote/AA
P1
P4
OCR (pmol/min/Norm. Unit)
6
4
2
0
0 20 40 60 80
Time (minutes)

Figure. S2

A

GEO study	Cell lines	Sample size	PMID
GSE9709	human iPSCs from neonetal skin derived cells(hiPSCs)	3	19383391
	human neonatal dermal fibroblast(hDF)	2	
GSE16963	iPSC of Human Third Molars	4	20595386
	human Third Molas cell line	2	

B

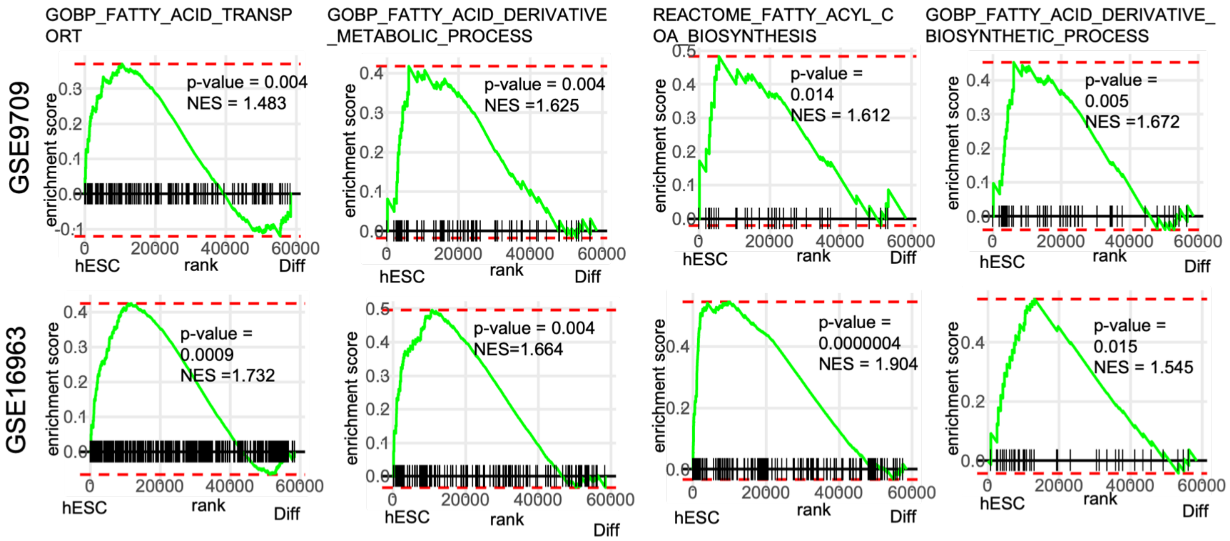

C

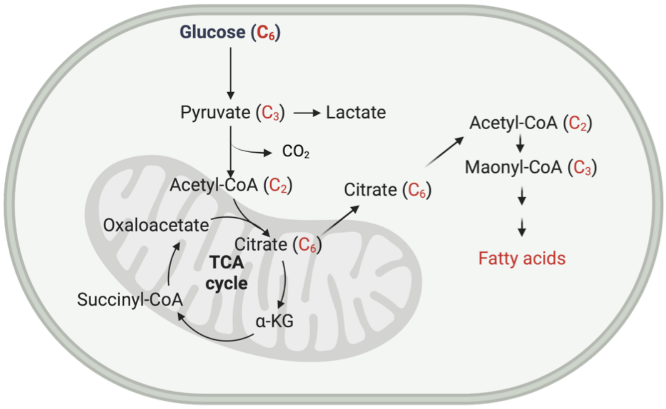

D

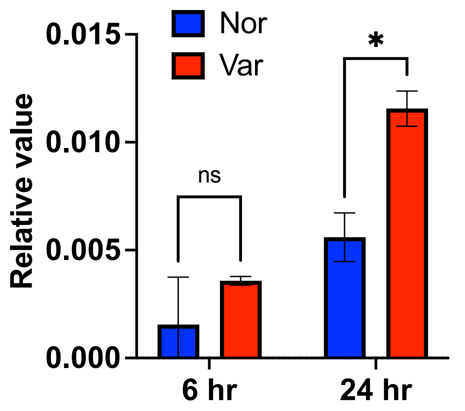

E

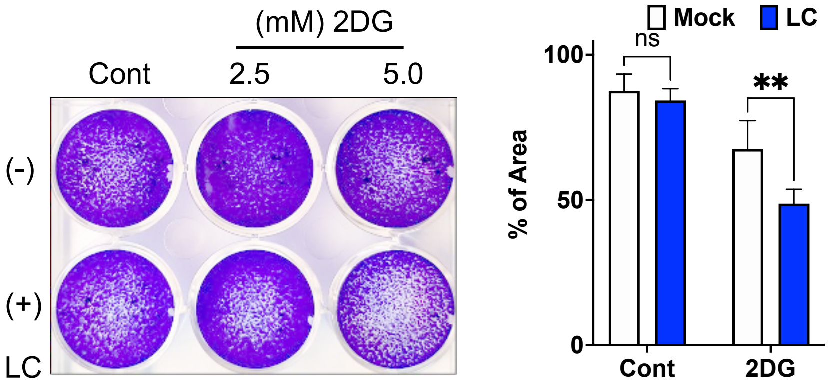

Figure. S3

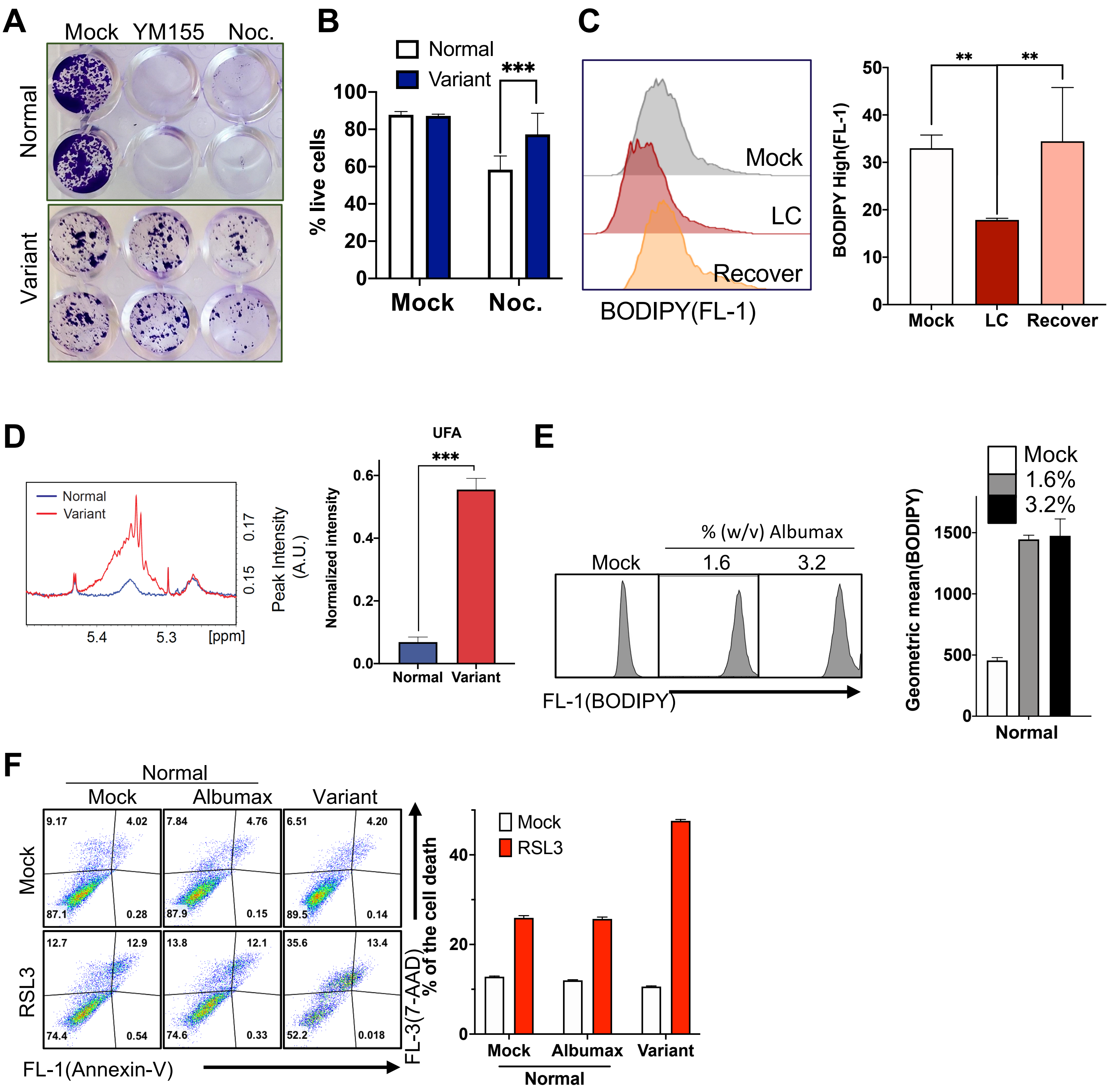

A
Mock YM155 Noc.
Normal
Variant
B
Normal
Variant
***
% live cells
100
80
60
40
20
0
Mock
Noc.
C
Mock
LC
Recover
BODIPY(FL-1)
**
**
BODIPY High(FL-1)
50
40
30
20
10
0
Mock
LC
Recover
D
Normal
Variant
0.17
0.15
Peak Intensity (A.U.)
5.4
5.3
[ppm]
UFA
***
Normalized intensity
0.6
0.4
0.2
0.0
Normal
Variant
E
% (w/v) Albumax
Mock
1.6
3.2
FL-1(BODIPY)
Mock
1.6%
3.2%
Geometric mean(BODIPY)
1500
1000
500
0
Normal
F
Normal
Mock
Albumax
Variant
Mock
RSL3
9.17
4.02
87.1
0.28
7.84
4.76
87.9
0.15
6.51
4.20
89.5
0.14
12.7
12.9
74.4
0.54
13.8
12.1
74.6
0.33
35.6
13.4
52.2
0.018
FL-1(Annexin-V)
FL-3(7-AAD)
% of the cell death
Mock
RSL3
40
20
0
Mock
Albumax
Variant
Normal

Figure. S4

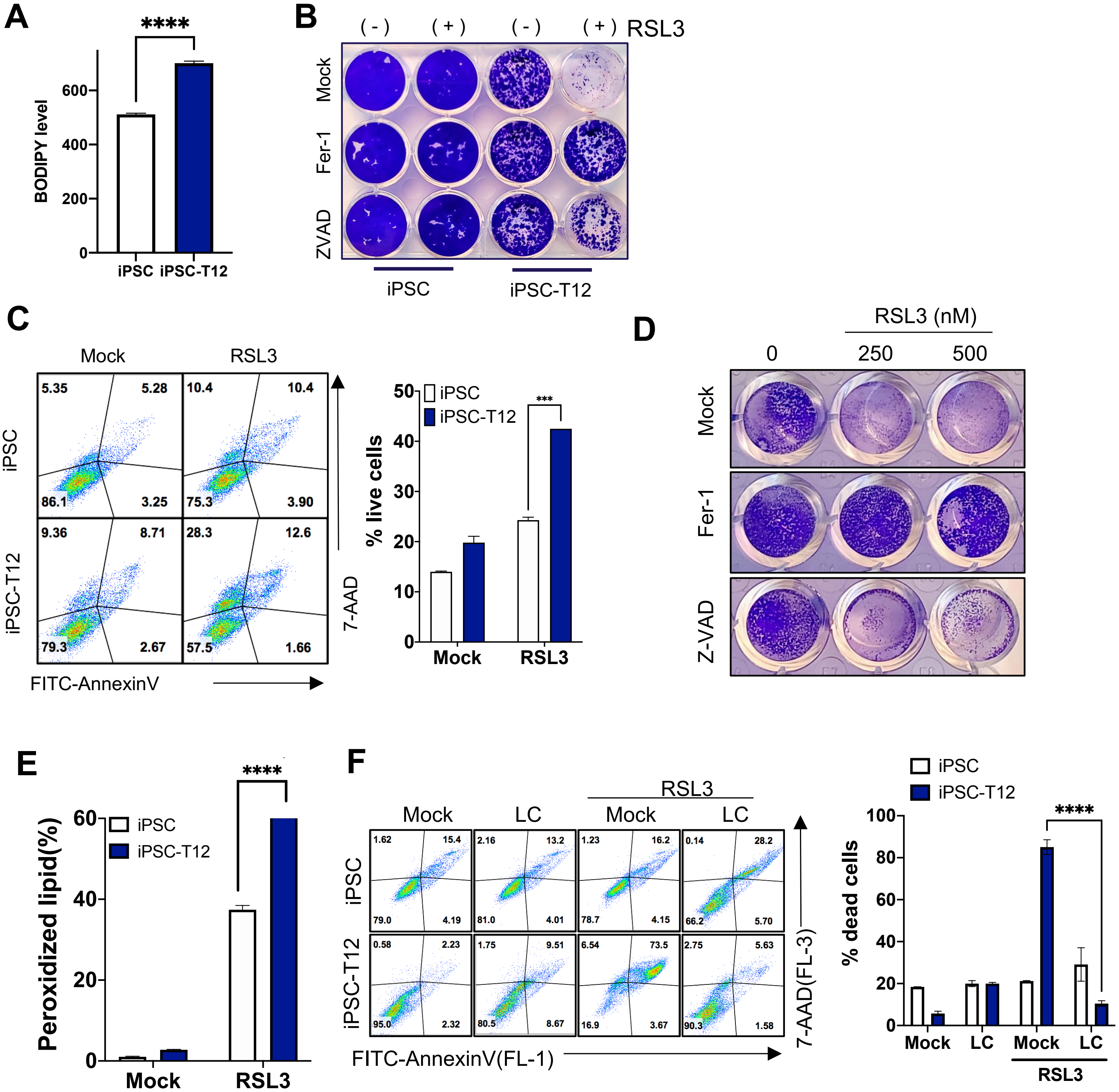

A
****
BODIPY level
600
400
200
0
iPSC
iPSC-T12
B
(-)
(+)
(-)
(+)
RSL3
Mock
Fer-1
ZVAD
iPSC
iPSC-T12
C
Mock
RSL3
iPSC
iPSC-T12
5.35
5.28
86.1
3.25
10.4
10.4
75.3
3.90
9.36
8.71
79.3
2.67
28.3
12.6
57.5
1.66
FITC-AnnexinV
7-AAD
% live cells
iPSC
iPSC-T12
***
50
40
30
20
10
0
Mock
RSL3
D
RSL3 (nM)
0
250
500
Mock
Fer-1
Z-VAD
E
Peroxidized lipid(%)
iPSC
iPSC-T12
****
60
40
20
0
Mock
RSL3
F
RSL3
Mock
LC
Mock
LC
iPSC
iPSC-T12
1.62
15.4
79.0
4.19
2.16
13.2
81.0
4.01
1.23
16.2
78.7
4.15
0.14
28.2
66.2
5.70
0.58
2.23
95.0
2.32
1.75
9.51
80.5
8.67
6.54
73.5
16.9
3.67
2.75
5.63
90.3
1.58
FITC-AnnexinV(FL-1)
7-AAD(FL-3)
iPSC
iPSC-T12
% dead cells
****
100
80
60
40
20
0
Mock
LC
Mock
LC
RSL3

Figure. S5

A

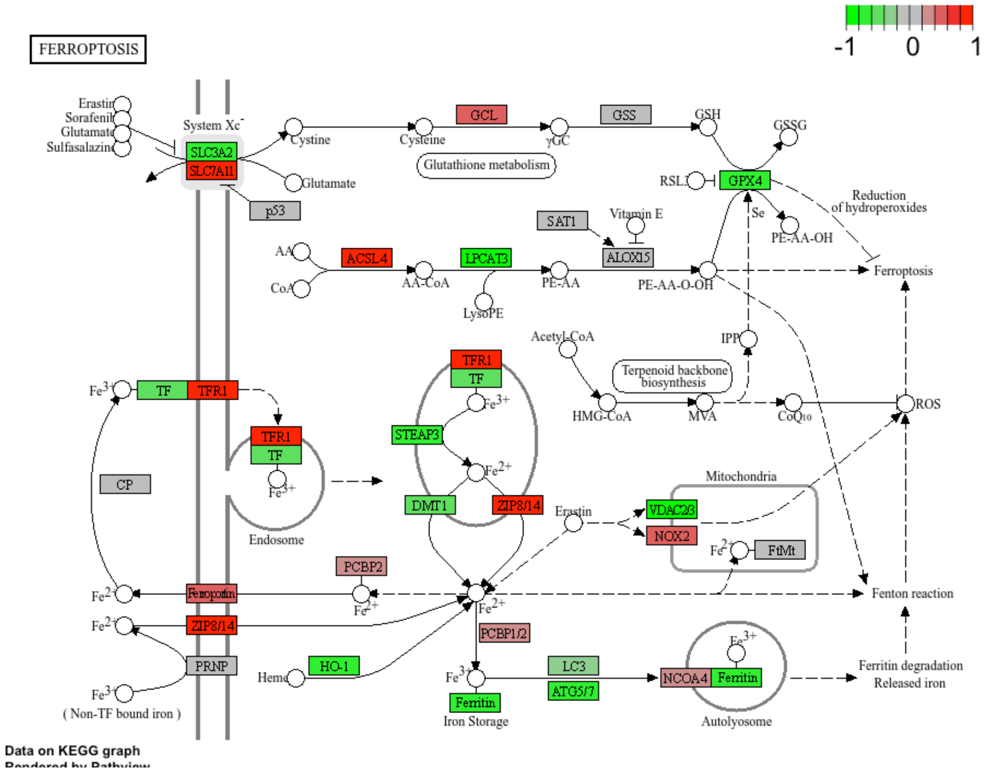

B

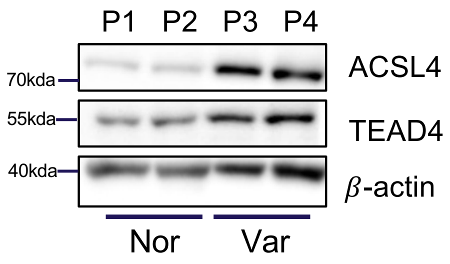

C

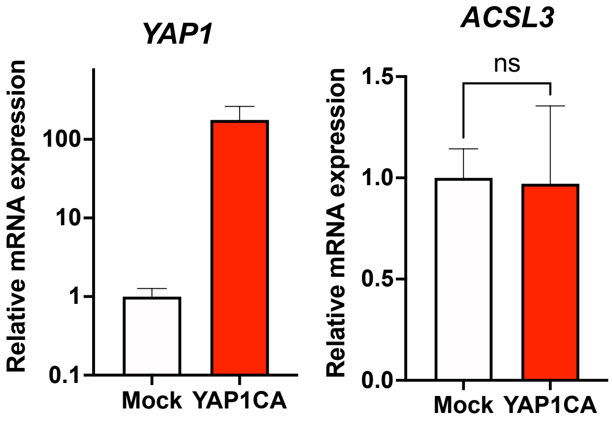

D

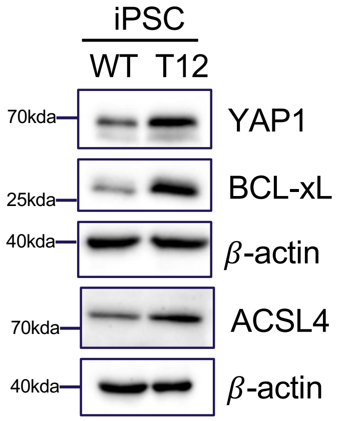

Figure. S6

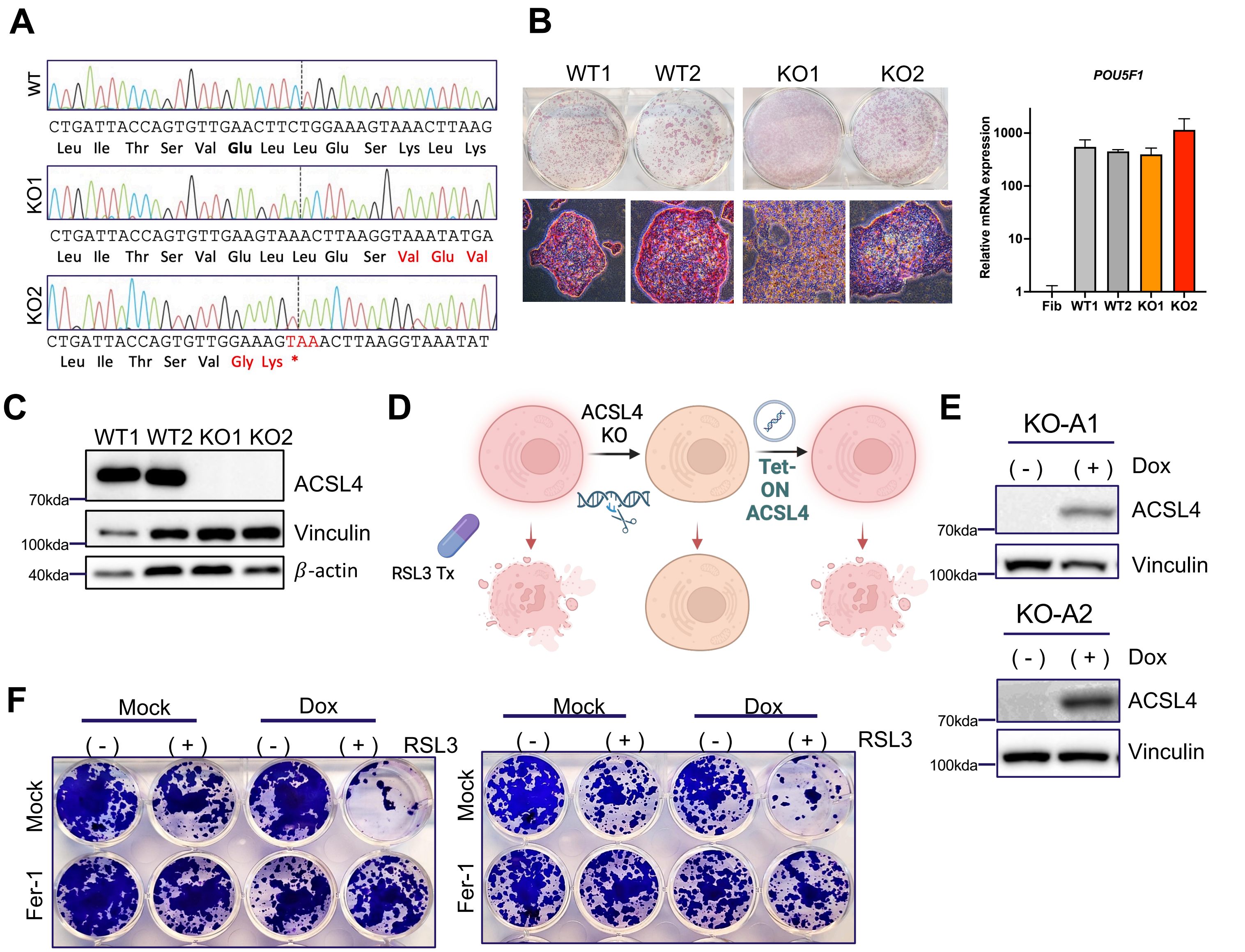
A
WT
CTGATTACCAGTGTTGAACTTCTGGAAAGTAAACTTAAG
Leu Ile Thr Ser Val Glu Leu Leu Glu Ser Lys Leu Lys
KO1
CTGATTACCAGTGTTGAAGTAAACTTAAGGTAAATATGA
Leu Ile Thr Ser Val Glu Leu Leu Glu Ser Val Glu Val
KO2
CTGATTACCAGTGTTGGAAAGTAAACTTAAGGTAAATAT
Leu Ile Thr Ser Val Gly Lys *
B
WT1
WT2
KO1
KO2
POU5F1
Relative mRNA expression
1000
100
10
1
Fib
WT1
WT2
KO1
KO2
C
WT1 WT2 KO1 KO2
ACSL4
70kda
Vinculin
100kda
β-actin
40kda
D
ACSL4 KO
Tet-ON ACSL4
RSL3 Tx
E
KO-A1
(-) (+) Dox
ACSL4
70kda
Vinculin
100kda
KO-A2
(-) (+) Dox
ACSL4
70kda
Vinculin
100kda
F
Mock
Dox
(-) (+) (-) (+) RSL3
Mock
Fer-1
Mock
Dox
(-) (+) (-) (+) RSL3
Mock
Fer-1

Figure. S7

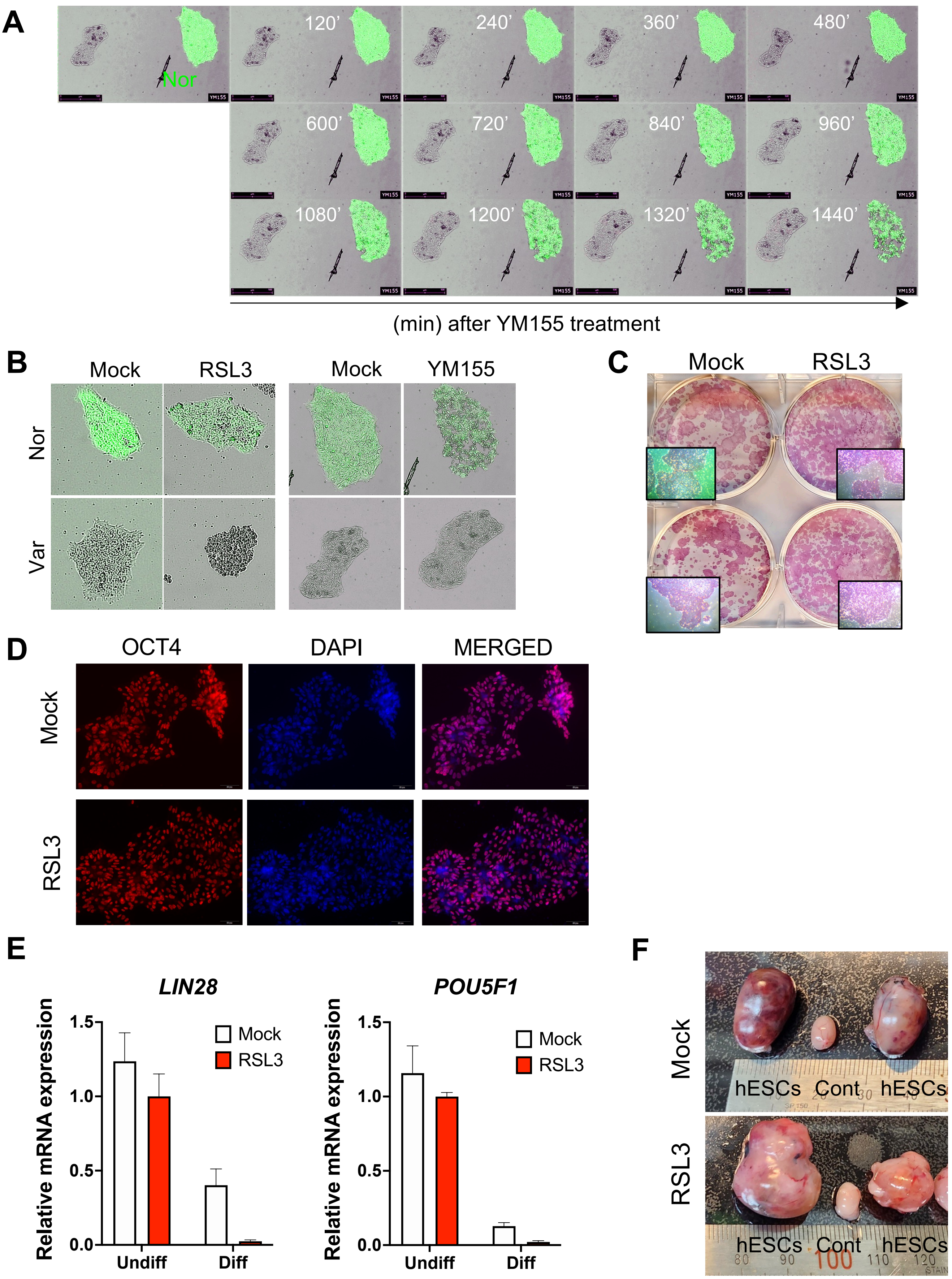
A
Nor
120'
240'
360'
480'
600'
720'
840'
960'
1080'
1200'
1320'
1440'
(min) after YM155 treatment
B
Mock
RSL3
Mock
YM155
Nor
Var
C
Mock
RSL3
D
OCT4
DAPI
MERGED
Mock
RSL3
E
LIN28
Relative mRNA expression
1.5
1.0
0.5
0.0
Mock
RSL3
Undiff
Diff
POU5F1
Relative mRNA expression
1.5
1.0
0.5
0.0
Mock
RSL3
Undiff
Diff
F
Mock
hESCs
Cont
hESCs
RSL3
hESCs
Cont
hESCs

Figure. S8

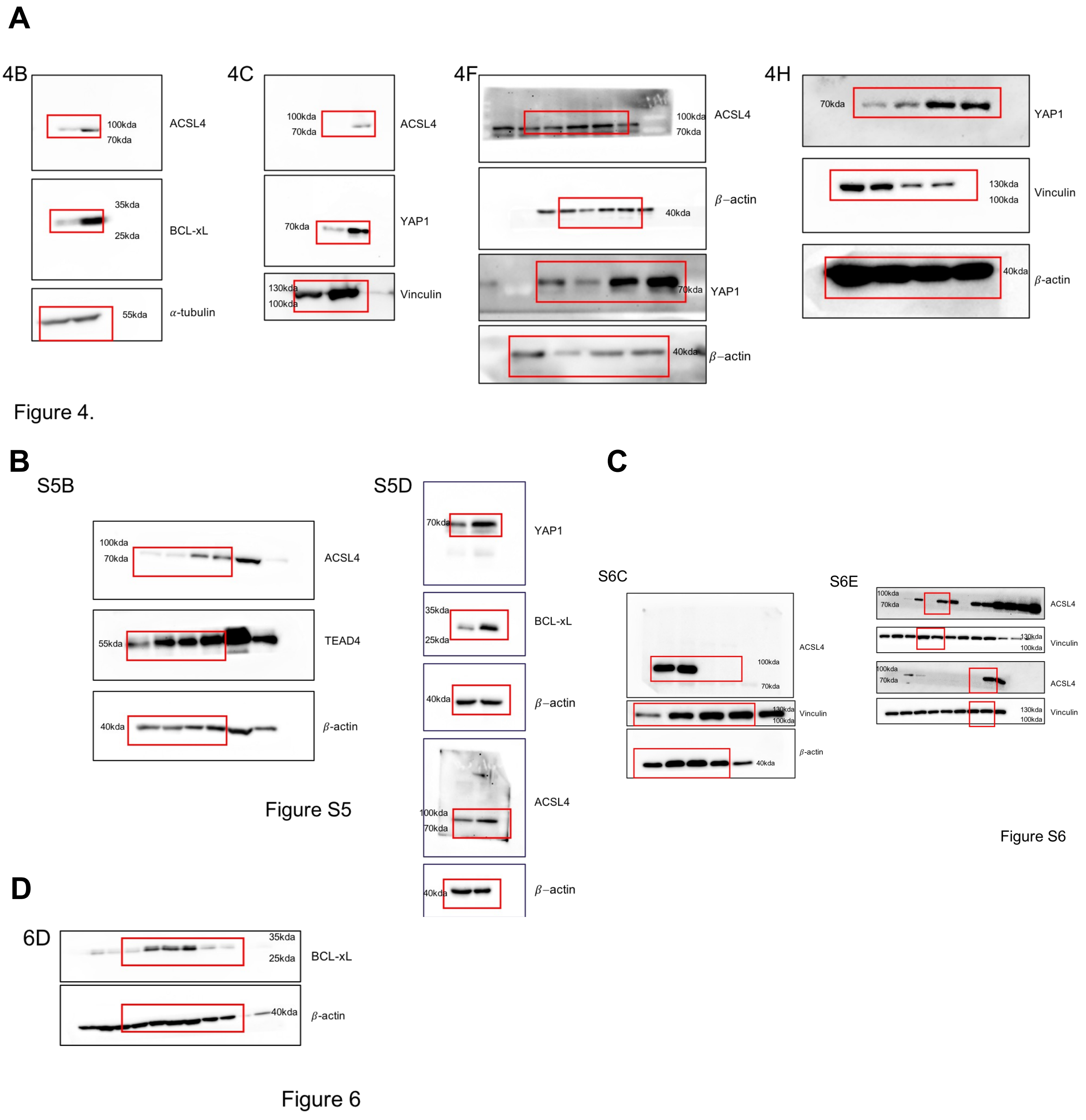
A
4B
100kda
70kda
ACSL4
35kda
25kda
BCL-xL
55kda
α-tubulin
4C
100kda
70kda
ACSL4
70kda
YAP1
130kda
100kda
Vinculin
4F
100kda
70kda
ACSL4
40kda
β–actin
70kda
YAP1
40kda
β–actin
4H
70kda
YAP1
130kda
100kda
Vinculin
40kda
β-actin
Figure 4.
B
S5B
100kda
70kda
ACSL4
55kda
TEAD4
40kda
β-actin
Figure S5
S5D
70kda
YAP1
35kda
25kda
BCL-xL
40kda
β–actin
100kda
70kda
ACSL4
40kda
β–actin
C
S6C
ACSL4
100kda
70kda
130kda
100kda
Vinculin
β-actin
40kda
S6E
100kda
70kda
ACSL4
130kda
100kda
Vinculin
100kda
70kda
ACSL4
130kda
100kda
Vinculin
Figure S6
D
6D
35kda
25kda
BCL-xL
40kda
β-actin
Figure 6

Table S1 Metabolite of Normal and Variant PSCs detected by lipidomics.

Metabolite name	PUFA lipid	Nor_1	Nor_2	Nor_3	Var_1	Var_2	Var_3	P value	Log2 fold ch	-log p value
**PC O-36:4|PC O-16:0_20:4**	o	1.09718024	0.94441614	0.95840362	0.5283616	0.568810898	0.492658	0.16883236	-0.5751174	0.77254432
**PG 44:12|PG 22:6_22:6**	o	0.88414902	1.14576762	0.97008337	0.5767883	0.555567085	0.528787	0.21331763	-0.5343192	0.67097325
**PC O-36:5|PC O-16:1_20:4**	o	1.15062004	0.87355551	0.97582445	0.58330455	0.595467812	0.540684	0.23034884	-0.494879	0.63761397
**PS 38:3|PS 18:1_20:2**	o	1.13076658	0.9363028	0.93293062	0.60288446	0.771836719	0.549885	0.16121583	-0.4261238	0.79259231
**PE 36:4|PE 16:0_20:4**	o	1.04761152	0.95394796	0.99844052	0.72016299	0.664101738	0.687076	0.23124388	-0.3334865	0.63592976
**PE 36:5|PE 16:1_20:4**	o	1.1438452	0.87301365	0.98314116	0.6984779	0.651896422	0.735768	0.26233106	-0.3745676	0.58115028
**PE 40:7;O|PE 20:4_20:3;O**	o	1.07128028	0.89795765	1.03076207	0.64830037	0.744892758	0.709462	0.35455026	-0.2852369	0.45032219
**PC O-38:5|PC O-18:1_20:4**	o	1.05126792	0.90347864	1.04525344	0.70565691	0.797639002	0.772821	0.4343541	-0.2022678	0.36215608
**PE O-38:3|PE O-16:0_22:3**	o	1.04875332	0.96095303	0.99029365	0.84294209	0.836503475	0.825427	0.21167076	-0.1752484	0.67433914
**PC O-38:2|PC O-16:0_22:2**	o	0.9968784	0.96267568	1.04044592	0.8438794	0.881936436	0.90448	0.51683867	-0.0875502	0.286645
CL 66:1|CL 16:0_16:0_16:0_18:1		0.99951161	1.00826706	0.99222133	0.05659647	0.057702257	0.08451	0.21264303	-1.4445331	0.67234885
DLCL 32:0		0.84840297	1.25295062	0.89864641	0.10346937	0.061638074	0.036325	0.16634762	-1.5671171	0.7789834
CL 68:2|CL 16:0_18:1_16:0_18:1		1.07317202	1.04085513	0.88597285	0.16080102	0.179896543	0.134321	0.12627252	-1.3702087	0.89869115
CL 68:2|CL 16:0_16:0_18:1_18:1		1.01046677	1.04801531	0.94151793	0.18401196	0.198235413	0.143517	0.16650356	-1.221896	0.77857648
PA 30:0|PA 14:0_16:0		1.16360348	0.92682458	0.90957194	0.26862914	0.22626223	0.281053	0.1488906	-1.158742	0.82713273
PA 30:1|PA 14:0_16:1		0.9892041	0.98516114	1.02563475	0.25307747	0.23919493	0.289882	0.24693987	-0.9642679	0.60740879
PA 32:1|PA 14:0_18:1		1.11396835	0.93885094	0.9471807	0.25538101	0.262550354	0.367686	0.17415062	-1.0715581	0.75907497
CL 70:2|CL 16:0_18:1_18:0_18:1		1.01012836	1.11234229	0.87752935	0.38597477	0.378137137	0.36646	0.09823809	-0.9555682	1.00772009
PMeOH 28:0|PMeOH 14:0_14:0		0.93357838	1.03886877	1.02755285	0.48997148	0.590009986	0.154964	0.28167134	-0.489393	0.55025733
PC 28:0|PC 14:0_14:0		0.99238746	0.99408512	1.01352742	0.43298674	0.457201754	0.460179	0.23909514	-0.6463532	0.62142925
PG 30:0|PG 14:0_16:0		1.17662969	0.88733988	0.93603043	0.44388075	0.464235767	0.455608	0.1724011	-0.7474306	0.76345997
PS 30:0|PS 14:0_16:0		1.02292624	0.9973015	0.97977226	0.46419656	0.465203502	0.444745	0.19066991	-0.6665331	0.71971784
CL 66:2|CL 16:0_16:1_16:0_18:1		1.10733807	0.89117623	1.0014857	0.51165538	0.582981552	0.32567	0.25316271	-0.5161673	0.59660027
PA 34:1|PA 16:0_18:1		1.16356364	0.98734984	0.84908651	0.42732519	0.456176756	0.544797	0.07662131	-0.896983	1.11565044
PE 30:0|PE 14:0_16:0		0.91773093	0.95997565	1.12229342	0.43161783	0.571451003	0.449844	0.46011486	-0.4062254	0.33713374
PE 28:0|PE 14:0_14:0		0.95211639	1.00536714	1.04251647	0.53903992	0.490106085	0.426535	0.2968785	-0.5031737	0.52742125
PE 30:1|PE 14:0_16:1		1.00732879	0.92258813	1.07008308	0.47224204	0.449813641	0.54872	0.33731755	-0.5391831	0.47196107
PE O-30:1|PE O-16:1_14:0		1.08351137	0.97854683	0.9379418	0.50885719	0.539146829	0.464507	0.13669755	-0.6392213	0.86423927
SM 40:2;O2		1.0422141	1.04398132	0.91380457	0.4986138	0.585086162	0.498369	0.1039768	-0.647638	0.98306356
PC O-32:1|PC O-16:0_16:1		1.09761436	0.93999412	0.96239151	0.48900271	0.55470267	0.542547	0.17829055	-0.6074481	0.74887168
PA 34:2|PA 16:1_18:1		0.99724477	1.06563767	0.93711756	0.52230339	0.468521261	0.635743	0.13714572	-0.6825623	0.86281773
PA 36:2|PA 18:1_18:1		1.07649271	1.05517695	0.86833034	0.49425958	0.503664728	0.634721	0.06855619	-0.7768005	1.1639533
PE 30:0|PE 14:0_16:0		0.98549389	1.18287228	0.83163383	0.63590074	0.599456872	0.432073	0.04467539	-0.6540385	1.34993161
SM 32:1;O2		1.11749576	0.99025464	0.8922496	0.59984137	0.544224867	0.584168	0.08406672	-0.6347052	1.07537589
SM 32:0;O2		1.108553	0.90217157	0.98927543	0.7018689	0.365457219	0.711869	0.28300536	-0.5524157	0.54820533
PE 32:1|PE 14:0_18:1		1.05163867	0.91183103	1.0365303	0.67718054	0.626702498	0.68205	0.33169504	-0.3316045	0.47926103
PS 32:1|PS 16:0_16:1		1.04341102	1.01531708	0.9412719	0.70385666	0.689837909	0.664828	0.09831982	-0.403314	1.00735893
SM 42:4;O2		1.1481515	0.8419395	1.00990899	0.64553389	0.730959413	0.749312	0.35341361	-0.3229596	0.45171673
PS 32:1|PS 14:0_18:1		1.12724926	0.95920688	0.91354386	0.68948198	0.72554722	0.722318	0.0923822	-0.4265709	1.03441169
PA 32:0|PA 16:0_16:0		1.15376342	0.91849994	0.92773665	0.6673162	0.682218171	0.795187	0.14199654	-0.4488639	0.84772224
DLCL 36:2|DLCL 18:1_18:1		1.04366186	0.93260766	1.02373047	0.65004735	0.801782365	0.757907	0.34591735	-0.2599872	0.46102765
Cer 32:1;O2|Cer 18:1;O2/14:0		1.13486898	0.95306346	0.91206756	0.71466096	0.806112189	0.733182	0.10226951	-0.3643957	0.99025383
Cer 32:1;O2|Cer 18:1;O2/14:0		1.1314745	0.96064593	0.90787957	0.74218375	0.79392157	0.738182	0.08179534	-0.360693	1.08727141
PC 30:1|PC 14:0_16:1		1.05703289	0.95742596	0.98554115	0.75978042	0.793556578	0.740663	0.19929018	-0.2511637	0.7005141
PE 32:1|PE 16:0_16:1		1.0647895	1.00232003	0.93289047	0.86504781	0.679721042	0.755209	0.13047777	-0.3235995	0.88446348
PC 30:1|PC 14:0_16:1		1.10170192	0.96476904	0.93352904	0.74660941	0.821644372	0.775142	0.10514398	-0.309175	0.97821559
PE O-32:0|PE O-16:0_16:0		1.04025244	1.05666043	0.90308713	0.80254392	0.821699304	0.815265	0.01444259	-0.3156152	1.84035496
PE 32:0|PE 16:0_16:0		0.9973479	0.99574736	1.00690474	0.85935225	0.866065043	0.773924	0.26114513	-0.1298456	0.58311806
SM 36:1;O2		1.06233372	0.95445665	0.98320963	0.83820784	0.879332975	0.854932	0.21348547	-0.1636633	0.67063169
PC 30:0|PC 14:0_16:0		0.98932731	0.98633682	1.02433587	0.88235929	0.922132518	0.877974	0.47125577	-0.0670959	0.32674332
**PE O-39:4|PE O-17:1_22:3**	o	0.9916347	1.06313224	0.94523307	1.15364769	1.185617686	1.151902	0.55159532	0.09173582	0.25837943
**PE 38:4|PE 18:0_20:4**	o	0.97996969	1.04688407	0.97314624	1.18741963	1.182529232	1.243116	0.36388165	0.1369799	0.43903985
**PC O-40:4|PC O-16:0_24:4**	o	1.02675383	0.92030564	1.05294052	1.28175332	1.190239361	1.221389	0.122979	0.27133647	0.91016906
**PE O-37:4|PE O-16:1_21:3**	o	1.068215	1.02553707	0.90624793	1.20615626	1.274056669	1.219155	0.61553302	0.1087252	0.21074864
**PE O-38:6|PE O-16:1_22:5**	o	1.04043647	0.98164634	0.97791719	1.20801231	1.16579661	1.343129	0.34026275	0.14409968	0.4681856
**PE 42:7|PE 20:1_22:6**	o	1.00539161	1.05492269	0.93968571	1.36566766	1.271480098	1.116355	0.40523926	0.21085602	0.39228849
**PI O-38:3|PI O-16:0_22:3**	o	1.13074378	0.92617835	0.94307787	1.32777193	1.293772392	1.221752	0.43119916	0.2082995	0.36532209
**PE O-42:5|PE O-18:2_24:3**	o	1.121766	0.99443445	0.88379955	1.34227922	1.339377019	1.18252	0.56517069	0.16764772	0.24782037
**Cer 42:5;O2|Cer 18:1;O2/24:4**	o	1.09924385	0.94746078	0.95329537	1.25674905	1.172261238	1.441209	0.48100882	0.13974163	0.31784696
**PE O-40:8|PE O-18:2_22:6**	o	0.99927859	1.08090915	0.91981225	1.14232932	1.441317273	1.299218	0.56371456	0.1671037	0.24894075
**PI 38:6|PI 18:2_20:4**	o	1.00315417	1.00372613	0.9931197	1.34520517	1.474537423	1.237025	0.24535448	0.34095736	0.610206
**PE 44:7;O|PE 22:4_22:3;O**	o	1.026957	1.02042662	0.95261638	1.38681801	1.367144499	1.303336	0.33034649	0.27134427	0.4810303
**PE 36:3|PE 16:0_20:3**	o	1.0252064	0.96002756	1.01476604	1.23958821	1.420878229	1.405248	0.22732287	0.30356398	0.64335687
**PS 40:5|PS 18:0_22:5**	o	1.04600886	1.00860762	0.94538351	1.30808082	1.406065217	1.379617	0.36644437	0.24782657	0.43599195
**PC O-38:3|PC O-18:0_20:3**	o	0.90076577	1.03708527	1.06214897	1.30421003	1.428605669	1.372957	0.13908024	0.38466628	0.85673458
**PC 36:5|PC 16:1_20:4**	o	1.13842163	0.91076535	0.95081302	1.35622496	1.430789689	1.329866	0.37001366	0.28218592	0.43178224
**PE O-38:4|PE O-16:1_22:3**	o	1.02542962	1.03737388	0.93719651	1.30280085	1.35111595	1.509637	0.39870137	0.21486228	0.39935228
**PE O-40:6|PE O-18:2_22:4**	o	1.00587227	1.02552223	0.9686055	1.48291229	1.530781537	1.152597	0.27234352	0.38616873	0.56488296
**PE O-42:6|PE O-20:2_22:4**	o	1.01156966	0.99800081	0.99042953	1.40241458	1.411930933	1.383396	0.23833871	0.33596148	0.62280542
**PI 39:4|PI 18:1_21:3**	o	1.05973574	0.92485411	1.01541015	1.32930114	1.46096371	1.407554	0.21819157	0.35434898	0.66116204
**PI O-36:3|PI O-16:0_20:3**	o	1.08369967	0.96721675	0.94908358	1.33147655	1.596698252	1.286267	0.35811151	0.33380568	0.44598171
**CL 72:7|CL 16:1_18:1_18:2_20:3**	o	1.04624184	0.98423085	0.96952731	1.44551299	1.499997056	1.290131	0.27710454	0.36224795	0.55735636

Table S2 Metabolite of ACSL4 KO and reconstruction (KO_Dox) detected by lipidomics.

Metabolite name	PUFA	KO1_1	KO1_2	KO1_3	KO1_Dox_1	KO1_Dox_2	KO1_Dox_3	P value	Log2 fold ch	-log p value
PE 40:7;O|PE 20:4_20:3;O	o	0.96310925	0.98104422	1.05584653	0.73813582	0.62398984	0.651108406	0.00180651	-0.5754476	2.743160765
PE 42:7;O|PE 20:4_22:3;O	o	1.02892431	0.96506032	1.00601537	0.72524993	0.65528423	0.689905793	0.00035407	-0.5350251	3.450908524
PE 42:8;O|PE 20:4_22:4;O	o	0.91925832	1.05335985	1.02738183	0.73316251	0.83302183	0.774264479	0.01192523	-0.3581773	1.923533123
PE 39:4|PE 18:1_21:3	o	1.27599096	0.89107148	0.83293756	0.5628728	0.58974755	0.636261021	0.04546031	-0.7459048	1.342367628
PE O-38:4|PE O-18:1_20:3	o	1.0627928	0.9082859	1.0289213	0.80859468	0.8787018	0.690071177	0.04545923	-0.3355975	1.342377891
PE O-36:6|PE O-16:1_20:5	o	0.93727464	1.03980972	1.02291564	0.76031262	0.81163846	0.813504985	0.00481137	-0.3306974	2.317730969
PI 38:4|PI 18:1_20:3	o	1.02569175	1.0199553	0.95435295	0.76111433	0.90961382	0.812849469	0.0248746	-0.2725427	1.604243857
PG 40:6|PG 18:1_22:5	o	1.02333837	1.00286487	0.97379676	0.89195013	0.95753098	0.83976583	0.04874543	-0.1577603	1.312066094
PS 38:5|PS 18:1_20:4	o	0.99349011	1.0140107	0.99249919	0.8706108	0.94160661	0.934168148	0.02310462	-0.1274283	1.636301163
FA 22:4		1.04879361	1.04034603	0.91086036	0.80654621	0.81385394	0.700788372	0.01720606	-0.3700988	1.764318687
PE 26:1|PE 8:0_18:1		1.04611032	1.17280124	0.78108844	0.43767211	0.49019442	0.431022714	0.00945432	-1.1425346	2.024369821
PE O-34:1|PE O-18:1_16:0		0.93733226	1.02755321	1.03511454	0.56428807	0.53145296	0.546784695	0.00016022	-0.8690465	3.795291761
PE O-30:1|PE O-16:1_14:0		1.04900406	0.95765939	0.99333655	0.48190101	0.57111926	0.651071837	0.00148169	-0.8159592	2.829243393
PC 36:1;O|PC 18:1_18:0;O		0.9937614	1.07637629	0.92986231	0.57645541	0.6544408	0.559954319	0.0014343	-0.7443176	2.843359982
PC O-34:0|PC O-18:0_16:0		1.06572463	1.0287611	0.90551427	0.61640033	0.62453123	0.583584875	0.00143451	-0.7174484	2.843296679
PE O-32:1|PE O-16:1_16:0		1.01635941	0.9916614	0.99197919	0.60273886	0.65141008	0.609710078	2.5518E-05	-0.6866698	4.593156566
PE O-36:1|PE O-20:1_16:0		0.93730111	1.13521685	0.92748203	0.59848409	0.68488492	0.583047901	0.00719547	-0.6846912	2.142941068
PE O-32:0|PE O-16:0_16:0		1.02044015	1.02085988	0.95869997	0.63370458	0.67417294	0.60467362	0.00023106	-0.6494642	3.636269506
FA 16:0;O		0.87732956	1.13640049	0.98626995	0.7251368	0.68945013	0.520005619	0.02242982	-0.6329328	1.649174264
PE 44:10		1.16121296	0.93559892	0.90318813	0.6850863	0.60705252	0.696426587	0.01715933	-0.5932345	1.76549956
PC 44:2|PC 18:1_26:1		1.03645729	1.04450089	0.91904182	0.61825996	0.71569295	0.658714332	0.00245132	-0.5902617	2.610599536
FA 30:7		1.09063304	1.03451076	0.8748562	0.67569199	0.70536549	0.61974503	0.00862616	-0.5843837	2.064182645
FA 30:7		0.98696003	1.09323763	0.91980234	0.71641092	0.71559584	0.590611198	0.00764884	-0.5687387	2.116404404
CL 62:1|CL 14:0_16:0_16:0_16:1		0.99868277	0.96498915	1.03632807	0.69847934	0.65832751	0.669819385	0.00016802	-0.5658825	3.774643055
PC 40:0|PC 16:0_24:0		1.12334434	1.0385029	0.83815277	0.71127523	0.68423858	0.664174982	0.02158378	-0.5425361	1.665872583
FA 16:3		1.09293925	1.02004036	0.88702039	0.68513084	0.69375629	0.702737733	0.00717935	-0.5272524	2.143915137
FA 30:8		0.95232335	1.06256498	0.98511167	0.71473034	0.69616567	0.686125634	0.00087292	-0.5166207	3.059024674
PC O-34:2|PC O-18:1_16:1		1.04530587	0.99849952	0.95619461	0.73251003	0.64993901	0.714756639	0.00111322	-0.5164941	2.953417358
FA 28:7		1.10060267	0.97479809	0.92459924	0.73185815	0.70156622	0.671931372	0.00565769	-0.5108985	2.247360548
PE O-34:2|PE O-18:2_16:0		1.08027318	0.9294452	0.99028162	0.71051739	0.7115852	0.692795741	0.00262538	-0.5043742	2.580807868
PC O-34:1|PC O-18:1_16:0		1.08266523	0.99579018	0.92154459	0.70535685	0.73676111	0.685539227	0.00400882	-0.4956968	2.396983937
FA 16:4		1.02522175	1.05698121	0.91779704	0.67510461	0.76157709	0.694759969	0.00428777	-0.4931329	2.367768037
PG 32:2|PG 16:1_16:1		0.94955457	1.12179472	0.92865071	0.7373514	0.80537192	0.590247025	0.03056942	-0.4920986	1.514712828
PE O-32:2|PE O-18:2_14:0		1.01979638	0.9423461	1.03785752	0.73840547	0.72075723	0.707224366	0.0008209	-0.4696715	3.085710749
PC 42:1|PC 16:0_26:1		1.04953156	1.06028797	0.89018047	0.73084745	0.75396346	0.682478297	0.00921784	-0.4690708	2.03537093
FA 16:3		0.97373358	1.05199394	0.97427247	0.7049713	0.74900407	0.715974401	0.00068941	-0.4673009	3.16152216
CL 66:3|CL 14:0_16:1_18:1_18:1		0.95578129	1.01849696	1.02572175	0.77231032	0.73370925	0.676965661	0.00155138	-0.4586601	2.809281952
FA 15:3		1.08670976	0.99428604	0.9190042	0.68119789	0.74134821	0.772784665	0.00841298	-0.4505242	2.075050281
MLCL 52:3|MLCL 16:1_18:1_18:1		1.09840318	1.01156346	0.89003336	0.8191156	0.76377997	0.612996666	0.03599688	-0.4501552	1.44373516
FA 15:4		1.03390773	1.03071422	0.93537805	0.71215698	0.759458	0.732812744	0.00164529	-0.4445583	2.783756792
PA 30:1|PA 14:0_16:1		0.99200015	1.04799253	0.96000733	0.86005529	0.59959556	0.752867303	0.03027175	-0.4392732	1.518962491
PE 34:1|PE 16:0_18:1		0.9921437	1.05565837	0.95219793	0.71887321	0.7987184	0.702301074	0.00356621	-0.4344726	2.447793273
PC O-36:3|PC O-18:2_18:1		1.1086992	1.03155031	0.85975049	0.74889373	0.78444243	0.694454114	0.03004044	-0.4293491	1.522293742
FA 15:3		1.03438234	0.99491963	0.97069802	0.70112088	0.78154088	0.771534822	0.00137405	-0.4123492	2.861998608
Hex3Cer 36:1;O2		1.09183623	0.98405459	0.92410918	0.80911628	0.71139679	0.750979776	0.01280784	-0.4013217	1.892524076
PE O-34:3|PE O-18:2_16:1		1.08308347	0.99388016	0.92303636	0.76565864	0.77761866	0.747342735	0.00740135	-0.3892243	2.130688963
PC O-36:1|PC O-20:1_16:0		1.01142044	1.02390448	0.96467508	0.78063118	0.73579038	0.793959903	0.00079999	-0.3768314	3.096915142
PE O-36:3|PE O-18:2_18:1		1.00622403	1.06922023	0.92455573	0.78792271	0.78568583	0.741888414	0.0068387	-0.3736406	2.165026762
PE O-36:2|PE O-16:1_20:1		0.98434765	0.99277451	1.02287784	0.78919421	0.75067743	0.781417741	0.00016722	-0.3700361	3.776700534
PC O-34:2|PC O-16:1_18:1		1.04376686	1.02138027	0.93485287	0.77036693	0.82414156	0.738704304	0.00587794	-0.3626446	2.23077497
PC O-32:1|PC O-16:0_16:1		1.05606794	0.98613597	0.95779609	0.77615182	0.78044821	0.777743394	0.00161554	-0.3619457	2.791683464
PC O-36:2|PC O-18:1_18:1		1.03356795	0.99905738	0.96737467	0.72411502	0.8158845	0.810886492	0.00362593	-0.3517579	2.440580838
PC 32:0|PC 16:0_16:0		1.09064229	1.0256538	0.88370392	0.81529405	0.78406822	0.759507458	0.02774174	-0.3468668	1.556866337
PC 32:1|PC 14:0_18:1		0.97833164	1.0743007	0.94736765	0.76968906	0.83465655	0.762929803	0.00906152	-0.3417349	2.042799133
CL 66:5|CL 16:1_16:1_16:1_18:2		1.01195208	0.99240399	0.99564393	0.85827086	0.76995671	0.739707013	0.00428682	-0.3413333	2.36786475
PC 28:0|PC 14:0_14:0		1.04284867	1.06370706	0.89344427	0.74694023	0.81909028	0.802447765	0.02202858	-0.3410021	1.657013447
PC 30:0;O|PC 16:0_14:0;O		1.11254339	0.98604895	0.90140766	0.79006543	0.85284656	0.729959604	0.04197831	-0.3383285	1.376975042
MLCL 54:3|MLCL 18:1_18:1_18:1		0.93417755	0.96838971	1.09743274	0.70327285	0.84970729	0.823128085	0.0362091	-0.3363619	1.441182219
PC 42:2|PC 16:0_26:2		0.92821274	1.08701025	0.98477701	0.79024867	0.82980076	0.757214346	0.01520679	-0.3356605	1.817962532
SM 44:5;O2		0.99863726	1.04067592	0.96068682	0.76112247	0.85917462	0.772498875	0.00634294	-0.3262651	2.197709143
PI 38:3;O|PI 20:3_18:0;O		1.00524327	1.0291517	0.96560503	0.84437901	0.8335318	0.717060287	0.01078144	-0.3249543	1.967323226
PS 36:1;O|PS 18:1_18:0;O		1.0247028	1.02877319	0.94652401	0.82408619	0.83043719	0.744621173	0.00647685	-0.3224424	2.188636102
PI 36:2|PI 18:1_18:1		1.05971273	1.00741489	0.93287238	0.747356	0.86621818	0.788175635	0.01705384	-0.3208766	1.768177833
PC 30:0|PC 14:0_16:0		1.00312495	1.05797546	0.93889959	0.80114037	0.87516055	0.771939511	0.01630239	-0.2932173	1.787748704
PC 34:1|PC 16:0_18:1		1.08654087	0.99864915	0.91480998	0.77036499	0.83308352	0.872068102	0.03889348	-0.2772329	1.410123158
CL 64:3|CL 14:0_16:1_16:1_18:1		1.04250799	0.92967617	1.02781584	0.90539793	0.78033689	0.801513722	0.03106277	-0.2704118	1.507759784
PE 36:3|PE 18:1_18:2		1.03457281	0.94944411	1.01598309	0.88123037	0.80476849	0.807082234	0.00938115	-0.2670327	2.027743935
Hex3Cer 38:1;O2		1.09134181	0.96337808	0.94528011	0.81485054	0.83273875	0.863551796	0.02761978	-0.2566194	1.558779837
PE 36:3|PE 18:1_18:2		1.00771796	0.98995809	1.00232396	0.78896603	0.89988044	0.835167441	0.00823193	-0.2492426	2.084498458
PE 34:2|PE 16:1_18:1		0.92647849	1.05124429	1.02227722	0.90806578	0.85949597	0.792120063	0.04386471	-0.228998	1.357884726
PC 38:2|PC 18:1_20:1		1.03549798	1.04448639	0.92001563	0.90588978	0.8424037	0.830665115	0.03895758	-0.2181739	1.409408056
SM 42:1;O2		0.9934898	1.02291491	0.98359529	0.88025479	0.89811809	0.822384908	0.00663863	-0.2060305	2.177921417
PC 34:0|PC 16:0_18:0		1.02769638	1.04994889	0.92235473	0.85816012	0.85858946	0.887668361	0.03130823	-0.2040015	1.504341536
PC O-36:0|PC O-20:0_16:0		0.98485496	1.04255608	0.97258895	0.82569009	0.84494586	0.935370804	0.0306557	-0.2031217	1.513488816
PC 32:2|PC 16:1_16:1		1.03812988	0.98614586	0.97572426	0.84733448	0.90680069	0.85349603	0.00837426	-0.2022227	2.077053545
PC 34:2|PC 16:1_18:1		0.99205249	1.03629466	0.97165286	0.86933109	0.82378534	0.917633795	0.0173154	-0.2004981	1.761567522
PC 50:5		1.05846308	0.91996349	1.02157343	0.84465435	0.87060916	0.900631937	0.04501522	-0.1976576	1.346640645
PE 32:1|PE 14:0_18:1		1.04861977	1.01968546	0.93169477	0.85501878	0.87181681	0.901494689	0.03030648	-0.1908159	1.51846454
PC 34:2|PC 16:1_18:1		1.03372427	1.03554819	0.93072754	0.87543423	0.86884672	0.891482807	0.02627548	-0.1867414	1.580449372
PE 34:2|PE 16:1_18:1		0.98009611	0.97973168	1.04017221	0.92982634	0.88131077	0.825192161	0.02884047	-0.1864319	1.539997665
PC 30:1|PC 14:0_16:1		0.95610208	1.06374011	0.98015781	0.84945049	0.8935195	0.917120516	0.04121924	-0.1734872	1.384899989
PE O-36:1|PE O-16:1_20:0		0.98719037	1.01340648	0.99940315	0.91051549	0.91375935	0.856730938	0.0060016	-0.1621882	2.22173306
PC 36:3|PC 18:1_18:2		0.96827742	1.03239912	0.99932346	0.93025112	0.86492623	0.897819835	0.01795685	-0.1557498	1.745769796
PS 32:1|PS 14:0_18:1		0.99399732	1.02655031	0.97945237	0.87269463	0.93894397	0.889026692	0.01479804	-0.1516477	1.829795928
CL 68:4|CL 16:1_16:1_18:1_18:1		0.98205143	1.00723525	1.01071332	0.96173751	0.96725289	0.925525042	0.03803133	-0.0717166	1.419858448
PC O-36:4|PC O-16:0_20:4	o	0.99122848	0.99105322	1.0177183	1.09315021	1.18038755	1.072903285	0.02773281	0.15766509	1.557006113
PI 38:5|PI 18:1_20:4	o	0.99983164	0.98012473	1.02004363	1.11318601	1.13495127	1.156463964	0.00136563	0.18252334	2.864665576
PE O-40:5|PE O-18:1_22:4	o	1.02010913	0.99765986	0.98223101	1.10198869	1.28362113	1.116715399	0.04759204	0.22335056	1.322465722
